# *Pseudomonas aeruginosa* core metabolism exerts a widespread growth-independent control on virulence

**DOI:** 10.1038/s41598-020-66194-4

**Published:** 2020-06-11

**Authors:** Stavria Panayidou, Kaliopi Georgiades, Theodoulakis Christofi, Stella Tamana, Vasilis J. Promponas, Yiorgos Apidianakis

**Affiliations:** 1 Infection and Cancer Laboratory, Department of Biological Sciences, University of Cyprus, Nicosia, Cyprus; 2 Bioinformatics Research Laboratory, Department of Biological Sciences, University of Cyprus, Nicosia, Cyprus

**Keywords:** Microbiology, Bacteriology

## Abstract

To assess the role of core metabolism genes in bacterial virulence - independently of their effect on growth - we correlated the genome, the transcriptome and the pathogenicity in flies and mice of 30 fully sequenced *Pseudomonas* strains. Gene presence correlates robustly with pathogenicity differences among all *Pseudomonas* species, but not among the *P. aeruginosa* strains. However, gene expression differences are evident between highly and lowly pathogenic *P. aeruginosa* strains in multiple virulence factors and a few metabolism genes. Moreover, 16.5%, a noticeable fraction of the core metabolism genes of *P. aeruginosa* strain PA14 (compared to 8.5% of the non-metabolic genes tested), appear necessary for full virulence when mutated. Most of these virulence-defective core metabolism mutants are compromised in at least one key virulence mechanism independently of auxotrophy. A pathway level analysis of PA14 core metabolism, uncovers beta-oxidation and the biosynthesis of amino-acids, succinate, citramalate, and chorismate to be important for full virulence. Strikingly, the relative expression among *P. aeruginosa* strains of genes belonging in these metabolic pathways is indicative of their pathogenicity. Thus, *P. aeruginosa* strain-to-strain virulence variation, remains largely obscure at the genome level, but can be dissected at the pathway level via functional transcriptomics of core metabolism.

## Introduction

*Pseudomonas* species include Gram-negative, rod-shaped, facultative anaerobic and polarly flagellated bacteria able to utilize a variety of organic compounds and colonize a wide range of niches^1^. Some *Pseudomonas* species can be pathogenic to humans, animals and plants^1–3^. While able to inhabit diverse environments, including soil and water, *P. aeruginosa* strains are common opportunistic human pathogens. They can also infect multiple model hosts, including insects, plants and mice using a remarkable variety of virulence factors^4–6^. In humans, *P. aeruginosa* causes acute infections in injured, burned and immunocompromised patients and persistent respiratory infections in individuals with cystic fibrosis^7–12^.

Previous comparative analyses of *P. aeruginosa* strain-specific genes revealed that different isolates tested share >90% of their gene content irrespective of the source (soil, wound, urinary tract, lung, blood or eyes) or the type of infection they were found to cause^3,13,14^. The high virulence of bacterial pathogens is usually attributed to the presence of pathogenicity islands, which are clusters of virulence-related genes often acquired through horizontal gene transfer^15,16^. The core genome of the highly virulent *P. aeruginosa* strain PA14 shares ~90% of its genes (as identical homologues) with the prototypic reference strain PAO1, but PA14 contains extra genes in genomic islands related to survival in diverse environments^17,18^. Despite the striking genomic similarity, *P. aeruginosa* strain-to-strain variation in virulence is vast^17,19,20^ and cannot be simply explained by the presence of virulence-related genes residing on pathogenicity islands^3^. This comes in contrast with genome decay often described in comparative genomic studies of pathogen adaptation to a host-associated lifestyle^6,21,22^.

The existence of multiple *P. aeruginosa* virulence factors and their direct regulators may indicate constant strain evolution at the virulence effector/regulator level or at a hierarchically higher gene network level. *P. aeruginosa* strains can evolve, for example, in the lung of cystic fibrosis patients, and some strains, such as CF5, are only weakly pathogenic in models of acute infection^13,19^. Nevertheless, extensive efforts to link gene content with pathogenicity of *P. aeruginosa* do not suffice to explain virulence by the mere presence or absence of known virulence factors^3,13,23^. Accordingly, *P. aeruginosa* pathogenicity has been characterized context-dependent, because genes required for pathogenicity in one strain may not necessarily contribute to virulence in other strains. We hypothesized that, in addition to gene presence, the expression of virulence factors and their direct or indirect regulators may explain the differences in pathogenicity among *Pseudomonas* strains.

In the current study, we compared 30 fully sequenced *Pseudomonas* genomes using large-scale comparisons at the protein sequence level. As a first step we aimed to re-examine the differences in the presence and absence of genes in relation to differences in pathogenicity among *Pseudomonas* strains by expanding our search to non-*P. aeruginosa* strains. We thus established an extensive set of known virulence factors (VFs), the expression rather than the gene presence of which correlated more reliably with the highly versus the lowly virulent strains. In addition, core metabolism gene expression correlated with pathogenicity, but at the pathway rather than at the gene level. Estimating the percentage of the core metabolism genes participating in the virulence of *P. aeruginosa* strain PA14, we found core metabolism genes to affect virulence at least as often as non-metabolic genes. Despite the known interconnectivity between bacteria core metabolism and growth^24^, most of core metabolism gene mutants tested were found defective in virulence and compromised in known virulence mechanisms, without being essential for growth in culture or in the host.

## Methods

### Bacterial strains

The catalog of the core metabolic genes of *P. aeruginosa* strain PA14 was produced using the KEGG Database in 2014^25^. The 553 metabolic gene mutants were picked from the 96-well plates of the publicly available PA14 Transposon Insertion Mutant Library, while the 95 non-metabolic gene mutants were randomly selected from sequential plates of the same library verifying that none of them is linked in the KEGG database with bacterial metabolism (http://ausubellab.mgh.harvard.edu/cgi-bin/pa14/home.cgi). The 30 wild type sequenced *Pseudomonas* strains used in this study are described in Supplementary Table 1.

### Intranasal mouse lung infection assay

The intranasal infection achieves the spreading of the bacteria from the upper airways to the intestine and low airways, thus mimics the pathology seen in acute bacterial pneumonia^26,27^. PA14 wild type and mutant strains were grown in LB liquid cultures overnight. Cultures were then diluted 1:100 and were grown over day to OD_600nm_: 3.0 (~3 × 10^9^ CFU/ml). One ml of each bacterial culture was centrifuged at 4610 rcf for 2 min and the supernatant was removed. The pellet was washed twice and finally diluted in sterile saline (0.9%). Mice were intranasally infected under very light anesthesia, as previously described^28,29^, by placing 10 μl of a bacterial suspension in each nostril (20 μl in total) to reach the desired infectious dose of 2 × 10^7^ CFUs per mouse. Mortality counts were taken every day for 7 days.

### Ethics statement

Animal protocols were approved by the Cyprus Veterinary Service inspectors under the license number CY/EXP/PR.L6/2018 for the Laboratory of Prof. Apidianakis at the University of Cyprus. The veterinary services act under the auspices of the Ministry of Agriculture in Cyprus and the project number is CY.EXP101. These national services abide by the National Law for Animal Welfare of 1994 and 2013 and the Law for Experiments with Animals of 2013 and 2017. All experiments were performed in accordance with these guidelines and regulations.

### Fly pricking assay

Male Oregon R flies were pricked in the thoracic cuticular epithelium with a tungsten needle dipped in a bacteria suspension, as previously described^30^. The infection mix contained 980 μl ddH_2_O, 10 μl 1 M MgSO_4_ and 10 μl of bacteria OD_600nm_: 3. Vials were transferred at 25 °C and survival was measured every day. Two vials of 20 flies each were used for each mutant.

### Fly wound colonization assay

20–25 Oregon R male flies were pump-injected dorsoventrally with 100 CFUs of a single bacterial strain grown at OD_600nm_: 3 and transferred into vials with fresh food at 25 °C, as previously described^30^. 24 hours later flies were ground in saline, plated on LB plates incubated for 16 hours at 37 °C before counting CFUs for each mutant strain in comparison to the wild type strain PA14.

### Fly feeding assay

Female Oregon R flies 3–7 days old were starved for 5–6 hours and then were transferred in vials containing a cotton ball soaked with 5 ml of the following oral infection mix: 0.5 ml of bacteria OD_600nm_: 3, 1 ml 20% sucrose and 3.5 ml ddH_2_Ο. Vials were transferred at 25 °C and survival was measured every day. Three vials with 10 flies each were used for each mutant.

### Fly survival

To determine the mortality rate of the infected flies, daily observation of the experimentations was necessary. Uninfected flies can live up to 60 days; however, infection using *P. aeruginosa* PA14 usually kills flies in less than 10 days. Fly infections were repeated in technical replicates two independent times for each bacterial strain for both assays (oral and wound infection). The fly survival was calculated based on the total number of flies surviving.

### Fly intestinal colonization assay

Oregon R female flies 3–7 days old, previously starved for 5–6 hours, were fed with the above described oral infection mix for one day. Flies were next transferred into 50 ml Eppendorf tubes with 12 holes (1.2 mm in diameter) on the lid, as previously described^31^. Flies were able to reach food (Whatman filter paper disc soaked with 200 μl of 4% sucrose and 10% LB set on the lid and covered with parafilm) only through the holes of the lid. Flies were transferred in clean 50 ml Eppendorf tubes every day for 3 days to avoid contamination. On the third day, plates were cultured with a solution of ground flies to count CFUs and observe the colonization ability of each mutant strain, compared to the wild type strain.

### Strain clustering based on pathogenicity

A survival graph was obtained for the flies infected by each bacterial strain and the survival curves were statistically analyzed using the Kaplan-Meier method^32^ using IBM SPSS. The resulting survival curves were compared pairwise in an all-against-all fashion, using a chi-square test (χ^2^). Applying the unweighted pair group method with the arithmetic mean (UPGMA) agglomerative clustering procedure^33^ (using the respective χ^2^-values as a pairwise distance) produces a tree-like representation for the strains analyzed. This “natural” hierarchical clustering was further used to separate *Pseudomonas* strains in three groups (corresponding to low, medium and high virulence) by cutting the tree in the second bifurcation from the root. Visual inspection of the Kaplan-Meier survival curves and the requirement that the well-characterized avirulent (i.e. CF5) or highly virulent strains (i.e. PA14) should be assigned as “low” and “high”, respectively, led to the designation of labels to the resulting clusters.

### Phylogenetic analysis of *Pseudomonas* strains

We produced a reliable phylogeny of the 30 studied *Pseudomonas* species/strains which was correlated with the pathogenicity scale. We followed the approach described in Duan *et al*.^34^, but *rpoB* and *rpoD* homologs could not be identified in the genome of *P. aeruginosa* 2192. Therefore, a phylogenetic tree was constructed using the sequences of the universally present gyrase B (*gyrB*) and 16S rRNA genes for the 30 studied species. *Escherichia coli* K12 and *Cellvibrio japonicus* strain Ueda107 were used as outgroups. Sequences were retrieved from the Pseudomonas Genome Database^35^ and the NCBI nucleotide database^36^ for the outgroups, and were multiply aligned using ClustalO^37^ with default parameters. The resulting multiple sequence alignments were concatenated using the MEGA software package^38^ and the data matrix produced was further analyzed with MrBayes v3.2^39^ (using the default parameters: lset nst = 6 rates = invgamma, mcmc ngen = 20000 samplefreq = 100 printfreq = 100 diagnfreq = 1000), which performs Bayesian inference of phylogeny. An essentially identical *Pseudomonas* phylogenetic tree was built with the complete gene matrix for the 29 species (i.e. excluding *P. aeruginosa* 2192) using the same procedure.

### Sequence analysis and clustering

Protein sequences encoded in the *Pseudomonas* strains of interest were downloaded from the Pseudomonas Genome Database^40^ and were internally codified following the style of the COGENT database for consistency and easy manipulation from computational tools^41^. Sequences were filtered with CAST^42^ (using default parameters) and converted in database records for the NCBI BLAST suite of tools^43^^,^^44^ as previous studies have shown that filtering sequences in regions of extreme amino acid composition can eliminate most of the false-positive results in BLAST searches without sacrificing sensitivity^45^. In this study, a new, more effective version of the CAST algorithm was used^46^ offering optimized performance for pan-genome analyses. We used a local installation of BLASTP for performing all-against-all pairwise comparisons (e-value threshold: 10^–6^; Composition Based Statistics: off; masking mode: off; all other parameters left to their default values). BLAST results were used for sequence clustering using the MCL software^47^ employed with the default parameters as suggested by the authors for the delineation of *Pseudomonas* protein families.

### Virulence factor (VF) selection

We collected annotated VFs encoded in the *P. aeruginosa* PAO1 genome based on the relevant section of the Pseudomonas Genome Database. Next, in order to obtain a comprehensive dataset of known VFs for most of the 30 studied species, an exhaustive literature search was manually realized in PubMed using keywords such as, “virulence factor”, “pathogenic capacity”, “secretion system”, in correlation with “*Pseudomonas”* species. Our list was further expanded by consulting the Virulence Factors Database (VFDB)^48^ as a complementary reference list. In this way, a list containing 254 virulence factors was created and VFs were then separated in five groups according to their function: type II secretion system (19 genes), type III secretion system (37 genes), type VI secretion system (18 genes), quorum sensing (44 genes) and other genes that did not enter any of these categories (136) (Supplementary Table 2).

### Metabolic genes

We identified the proteins annotated to participate in distinct metabolic pathways and categories based on the KEGG database (Kyoto Encyclopedia of Genes and Genomes, www.kegg.jp ^25^. For the assessment of RNAseq transcriptomics data (see below) against metabolic pathways, we deliberately used an independent pathway definition provided by BioCyc^49^, in order to avoid any potential biases due to our initial selection of metabolic genes.

### Phylogenetic profiles of *Pseudomonas* VFs and metabolic genes

We did not rely on external resources to infer homology among proteins encoded in the genomes of interest, but we used the bidirectional-best hit (BBH) as a more reliable approach. Briefly, parsing the all-against-all BLASTP results with custom-developed software, we identified cases where a protein *protA* from “Genome A” has *protB* as a best hit (according to the BLASTP score) in “Genome B”, which in turn has *protA* as its best hit in “Genome A”. This procedure rapidly identifies cases of 1–1 orthologs, with the drawback that some subtle cases (e.g. where recent gene duplication has occurred) may be missed^50^. Moreover, cases of wrongly predicted genes are expected to result in additional proteins that seem to be absent from some genomes of interest. In order to alleviate this drawback, all proteins of interest that appeared to be absent from particular *Pseudomonas* strains after the BBH analysis were further queried using translated BLAST searches (TBLASTN) against the respective target genomes and if reliable hits were found they were recorded as “present”. Phylogenetic profiles for *Pseudomonas* VFs and metabolic proteins were coded in Presence-Absence (PA) tables using a simple +1/−1 scheme (present/absent). PA tables were further visualised using the MeV software^51^ multiple array viewer feature and were subject to clustering using the k-means method in the R environment^52^. These clusters were then compared to the groups obtained by the pathogenicity ranking using the Adjusted Rand Index (ARI)^53,54^ as implemented in the mclust R package. The ARI is a measure for the chance grouping of elements between two clusterings accompanied by a measure of the statistical significance of the overlap of the groups compared.

### Bacterial RNA isolation for RNA Seq

We extracted RNA from LB cultures at OD_600nm_ 1 and 3 in biological replicates using the QIAzol lysis reagent (Qiagen). The quantity of the bacterial RNA samples was measured on a NanoDrop ND1000 system and their quality analyzed on the Agilent 2100 Bioanalyzer system with the Agilent RNA 6000 Nano kit protocol (Agilent Technologies), according to manufacturer’s instructions. Bacterial RNA samples with RNA Integrity Number (RIN) >7 were chosen for mRNA enrichment. The MICROBExpress Bacterial mRNA Enrichment Kit protocol (ThermoFisher Scientific) was performed on 5–10 μg of bacterial RNA, according to the manufacturer’s instructions. The quality and quantity of enriched mRNA were measured using the NanoDrop and the Bioanalyzer systems. After the assessment of the enriched bacterial mRNAs, libraries were prepared using the Ion Total RNA-Seq Kit v2 protocol and reagents (ThermoFisher Scientific) according to the manufacturer’s instructions.

The enriched mRNAs were fragmented, reverse transcribed and amplified after the addition of a specific (~10 bp) barcode to each sample. The quantity and quality of the prepared libraries were assessed on a Bioanalyzer using the DNA High Sensitivity Kit, according to the manufacturer’s instructions. All libraries were diluted to 1 nM and subsequently pooled together in pools of eight. 40 pM of pooled libraries were further processed for templating and enrichment, and loaded onto the Ion Proton PI V2 chips (ThermoFisher Scientific). The procedure was performed on the Ion Chef System using the Ion PI IC200 Chef kit, protocol and reagents (ThermoFisher Scientific), according to the manufacturer’s instructions. The sequencing of the loaded chips was performed using the Ion PI Sequencing 200 V3 kit on an Ion Proton system (ThermoFisher Scientific), according to the manufacturer’s instructions. Initial analysis, sample/barcode assignment, and genome mapping was performed on the Ion Proton server.

### Short read mapping

The RNA-Seq FASTQ files obtained after Ion Proton sequencing (publicly accessible through the Gene Expression Omnibus database, GEO Series accession number GSE142464 and the following link: https://www.ncbi.nlm.nih.gov/geo/query/acc.cgi?acc=GSE142464) were mapped using tophat2^55^ (https://ccb.jhu.edu/software/tophat/index.shtml), with default settings and using additional transcript annotation data for the *Pseudomonas aeruginosa* (UCBPP-PA14) genome (GCF_000014625.1_ASM1462v1_genomic.gff). Unmapped reads were submitted to a second round of mapping using Bowtie2^56^ (http://bowtie-bio.sourceforge.net/bowtie2/index.shtml) against the *Pseudomonas aeruginosa* (UCBPP-PA14) genome, with the very-sensitive switch turned on and merged with the initial mappings. Reads per gene were counted using featureCounts^57^ and those corresponding to rRNA genes were in silico depleted to avoid skewing the statistics of independent samples prior to processing with DESeq2^58^ for the detection of differentially expressed genes.

### Pathway enrichment analysis

Relevant gene lists were uploaded to BioCyc SmartTables. Pathway enrichment was performed using the respective BioCyc functionality implementing the Fischer exact test and the Parent-Child Intersection method that has been shown to be robust against false positives^59^. Multiple testing correction was performed with the Benjamini-Hochberg procedure. Adjusted p-values were considered significant when p_adj_ < 0.05.

### Cluster analysis of patterns of differential gene expression

Cluster analysis and visualization of patterns of up-/down-regulation was performed with ClustViz^60^ on a matrix where up-/down-regulation of each gene was represented with +1/−1, respectively. Zero values were used to denote the absence of significant differential expression.

### Minimal media assays

Bacteria that were grown in 3 ml LB O/N cultures, 500 μl were centrifuged at 8000 rpm for 2 min, the supernatant was removed, and the pellet was reconstituted and further diluted 1:100 in minimal media and incubated on a horizontal culture rotator at 37 °C. To prepare 50 ml of minimal medium solution we mixed 10 ml 5xM9, 100 μl 1 Μ MgSO_4_, 1 ml 20% glucose and 39 ml ddH_2_O. An alternative minimal medium solution was prepared by adding a final concentration of 5% ground and filtered fly extract to the above mix. For each mutant, two replicate cultures and three consecutive measurements at optical density OD_600nm_ were taken.

### Quantitative reverse transcription real-time PCR

RNA was extracted from log-phase bacteria grown in LB using the QIAzol Lysis Reagent. Briefly, 500 μl of bacteria OD_600nm_: 1.5 (~10^9^) were spun down at 8000 rpm for 3 minutes. The pellet was dissolved by adding 500 μl of QIAzol and by pipetting up and down at 60 °C for 10 minutes. Next, 100 μl CHCl_3_ was added and tubes were inverted for 15 seconds. After a 5 minute incubation at room temperature, the supernatant (300 μl) was selected in a new tube, by a full-speed centrifugation at 4 °C for 15 minutes and then mixed with 300 μl iso-propanol by inverting the tubes. The tubes were then let on the bench for 5 minutes and, after a step of full-speed centrifugation at 4 °C for 10 minutes, the pellets were washed out with 500 μl of 70% EtOH. Finally, after centrifugation at 4 °C for 3 minutes and air drying, pellets were resuspended in 20–50 μl RNase free H_2_O by pipetting and stored at −80 °C. Bacterial RNA was reverse transcribed into complementary DNA (cDNA) using the PrimeScript RT reagent Kit (Perfect Real Time) (Takara: RR037A) according to the manufacturer’s instructions. Real-time PCR was performed, using a Bio-Rad CFX1000 thermal cycler and Bio-Rad CFX96 real-time imager with primer pairs listed below and iQ SYBR green supermix (Bio-Rad).

**Primer sequences (5**′**-3**′**)**. “F” and “R” indicate the direction (forward or reverse) of each primer

***rplU*** F: ATGGCGAAGACGTGAAAATC, R: GAACTTGATGATGCGGACCT

***clpX*** F: GTTCGGTCTTATCCCCGAGT, R: AACAGCTTGGCGTACTGCTT

***exsC*** F: CACCGTTTCGATCTGCATTTCG, R: CGAGAATCTGCGCATACAACTG

***exoT*** F: GCCGAGATCAAGCAGATGAT, R: TTCGCCAGTCTCTCCTCTGT

***pa1L*** F: GGTTGCACCCAATAATGTCC, R: CCAATATTGACGCTGAACGA

***pilA*** F: CAGAGGCGACTGGTGAAATC, R: AGGGTAGAGTCAGCCGGAAT.

For each target gene two independent experiments were performed in biological triplicates. All samples were normalized to the expression of the control genes *rplU* and *clpX* via the Pfaffl method^61^.

### Swarming motility

Swarming motility was assessed using Petri dishes each containing exactly 20 ml of medium with 5 g/l Bacto-agar (Difco), 8 g/l Nutrient Broth (Difco) and 5 g/l Dextrose. Bacterial cultures with the strains of interest were grown overnight in LB medium and 2 μl from each culture was added at the center of a swarming plate. The plates stayed open until the droplet was fully absorbed by the agar and then incubated for 24 hours at 37 °C. The diameter of the swarming zone on the plate was measured, and photos were taken.

### Twitching motility

Twitching motility was assessed using Petri dishes containing 1.0% Bacto agar and 20 g/L LB broth. After the agar was solidified, the indicated strains were stabbed at the bottom of the plates with a sterile toothpick. The plates were incubated at 37 °C for 48 hours. The ability of the bacteria to adhere and form biofilms was examined by removing the agar, washing the unattached cells with water and staining the attached cells with crystal violet (1%). The stain solution was removed by carefully washing the plates with water. The diameter of the twitching zone on the plate was measured, and photos were taken.

## Results

### Pathogenicity-based classification of *Pseudomonas* strains

**Figure 1:**
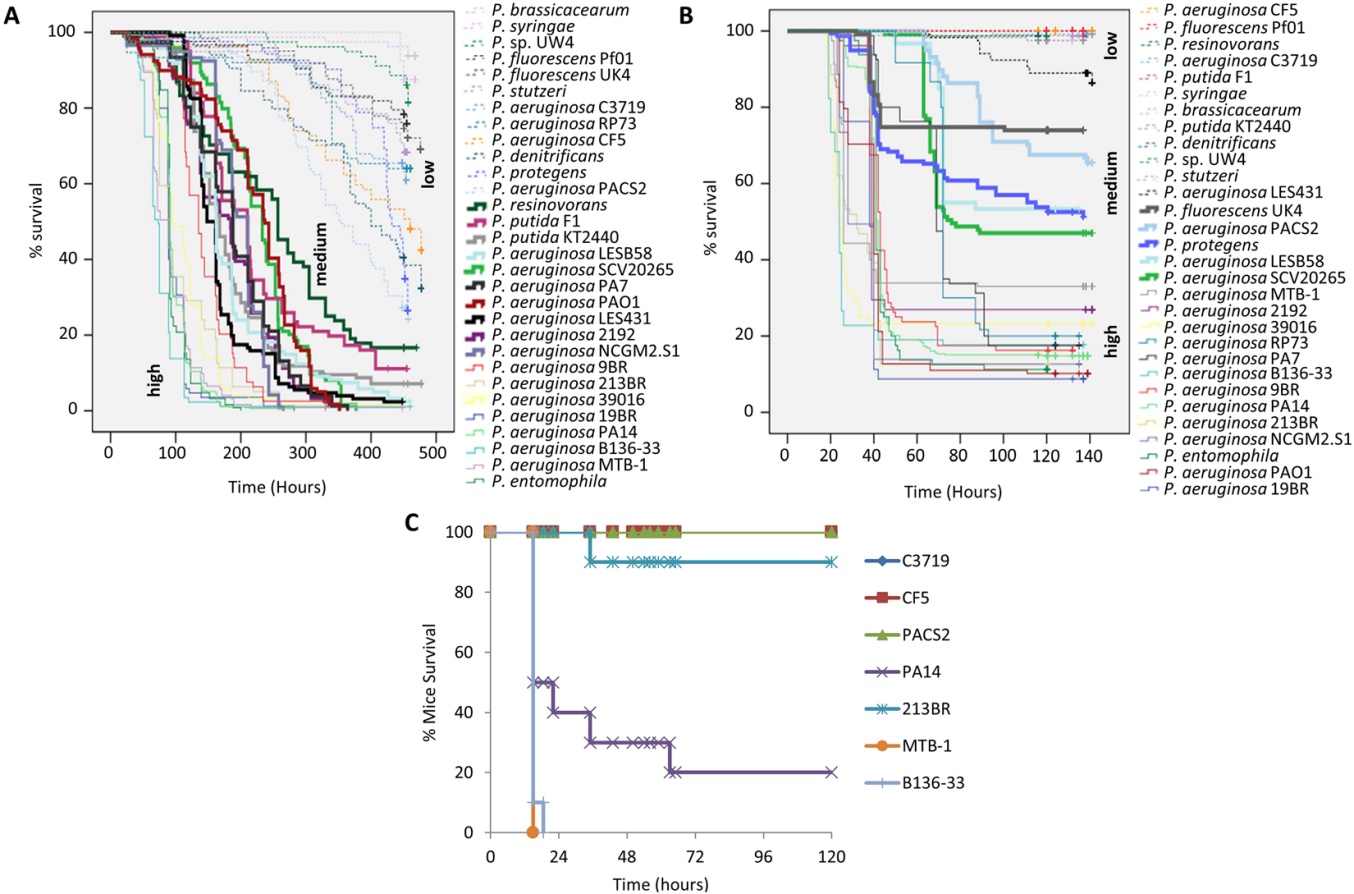
Comparative survival of all 30 *Pseudomonas* strains upon oral and wound infection in flies and lung infection in mice. (**A,B**) Three groups are distinguished, each representing species that are either high, medium or low virulent in fly oral (A) and wound (B) infection. Thin lines represent high virulent strains, thick lines represent medium virulent strains and dashed lines represent low virulent strains. Time is measured in hours. (**C**) Mouse survival (%) after intranasal mice infection with 3 lowly and 4 highly virulent *P. aeruginosa* strains. Twenty-microliter aliquots of a bacterial solution containing 2 × 10^7^ bacteria were administered intranasally to each mouse. Infected mice were monitored for 5 days (n = 10).

To correlate the pathogenicity of 30 fully sequenced *Pseudomonas* strains (18 *P. aeruginosa* and 12 other *Pseudomonas* strains; Supplementary Table 1), we examined their virulence in two distinct types of acute infection: an oral and a wound *Drosophila melanogaster* infection assay (Fig. 1A,B)^30,62^. Kaplan-Meier survival analysis with a log-rank test and pairwise comparison over strata was used to analyze the results of fly survival after the infection. Even though it is common to group bacterial strains based on visual inspection of their survival curves, this procedure is subjective. To objectively partition the strains under study in terms of pathogenicity, the 30 bacterial strains were classified in three groups (low, medium and high), according their effect on fly survival (Fig. 1A,B and Supplementary Table 1), using hierarchical clustering (see Materials and Methods; Supplementary Tables 3-4). Based on published data, PA14 and CF5 were used for both infection type assays to define the “high” and “low” virulence clusters, respectively^13,19^.

Non-*P. aeruginosa* strains were grouped in the “low” and “medium” virulence clusters, with a notable exception of *P. entomophila* presence in the “high” virulence cluster. *P. entomophila* is a known entomopathogenic bacterium, able to infect and kill insects, including *Drosophila*^63^. *P. aeruginosa* strains B136-33, MTB-1, PA14, 213BR, 19BR, 9BR and 39016 were consistently virulent, while strains CF5 and C3719 consistently low in virulence regardless of the infection assay. Some *P. aeruginosa* strains were less consistently grouped between assays, such as PAO1, which was grouped with the “high” and “medium” virulence cluster in the wound and oral infection assays, respectively. To validate and more rigorously select high and low in acute virulence strains we used a model of acute intranasal mouse lung infection. We assessed the mortality rate of four of the highly (B136-33, MTB-1, PA14, 213BR) and the three most lowly (C3719, CF5, PACS2) virulent in *Drosophila P. aeruginosa* strains. We found that all but one (213BR) of the 7 tested strains retained their virulence potential in the acute mouse lung infection model (Fig. 1C).

### Presence-Absence of single genes cannot explain *Pseudomonas* pathogenicity

We inferred a phylogenetic tree of all the 30 *Pseudomonas* strains based on the concatenated alignments of two core housekeeping genes, the 16 S rRNA and *gyrB* (Supplementary Fig. 1) in agreement with the trees constructed using four housekeeping genes or 1679 orthologs per Duan *et al*.^34^. Our results were also in agreement with previous observations that place *Pseudomonas* sp. UW4 within the *P. fluorescens* clade, even though phylogenetic analysis based on 16S rRNA sequences alone indicates a closer relationship to the *P. putida* clade.

**Figure 2:**
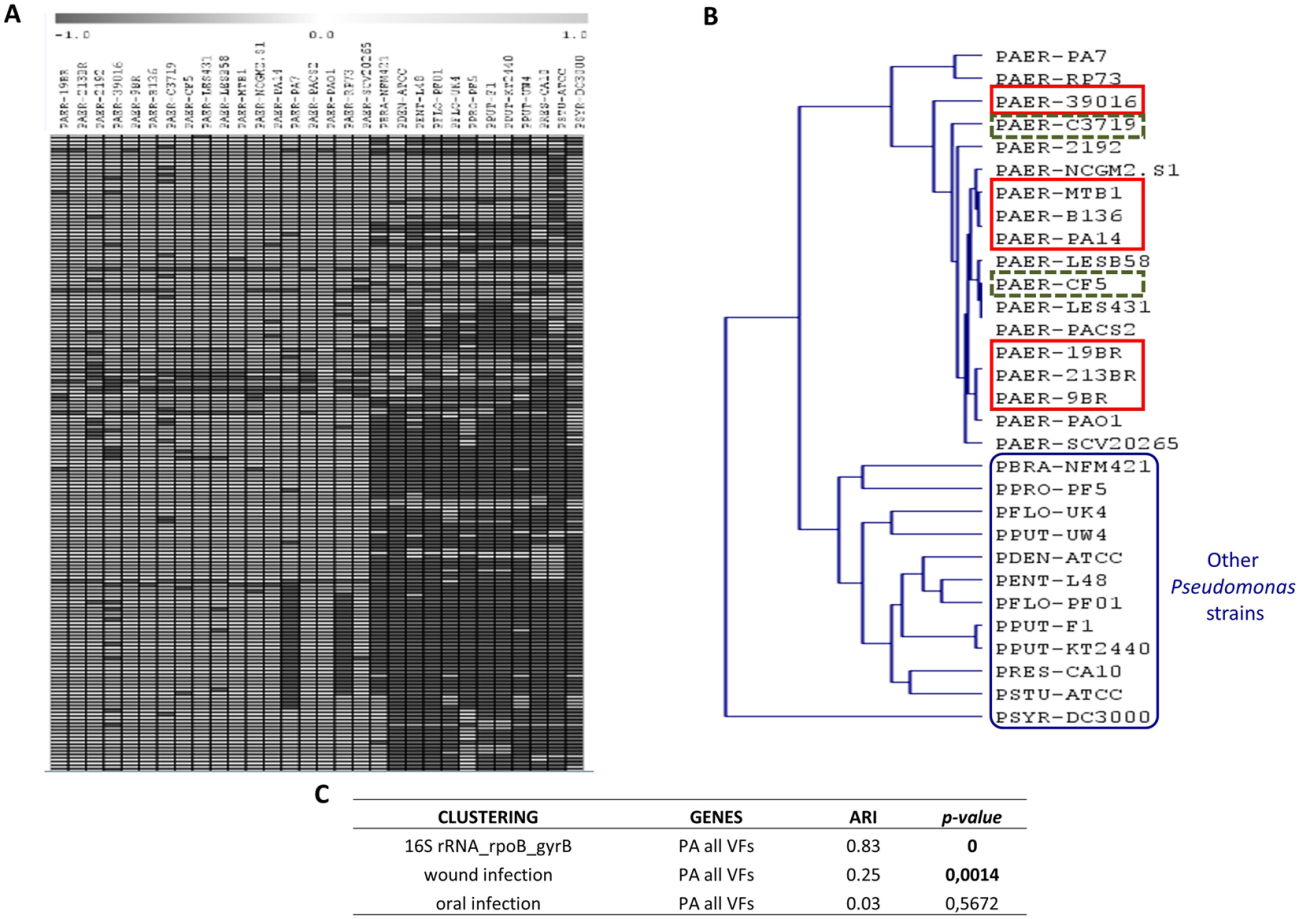
Virulence factor Presence-Absence follows phylogeny rather than pathogenicity. (**A**) Part of a multiple array viewer based on the Presence-Absence (PA) of virulence factors (VFs). Present genes are shown in light grey, absent genes are shown in dark grey. The 18 vertical lanes on the left correspond to the *18 P. aeruginosa* strains, while the next 12 lanes to the other *Pseudomonas* strains. (**B**) Clustering based on the presence/absence of virulence factors. The 2 lowly virulent in both fly infection assays *P. aeruginosa* strains are shown in dashed-line boxes; and the 7 high virulent in both assays *P. aeruginosa* strains in full-line boxes. (**C**) Phylogeny rather than pathogenicity of the 30 *Pseudomonas* spp. correlates best with Presence-Absence of virulence factors.

Next, we created a database of 254 virulence factors (VFs) previously found in the *Pseudomonas* species included in our analysis (see Methods). We classified these VFs according to their functions in five groups: type II secretion system (T2SS; 18 genes), type III secretion system (T3SS; 38 genes), type VI secretion system (18 genes), quorum sensing (44 genes) and other (136) (presented sequentially in the Supplementary Table 2). For each category a BBH BLASTP search allowed us to detect the phylogenetic profiles of VFs across the *Pseudomonas* species. Five multiple array viewers (one for each functional category) were obtained indicating the presence-absence of VFs. A sixth multiple array viewer was obtained by merging all VFs together (Fig. 2A). All VFs found to be absent in a particular genome were manually re-examined with a TBLASTN analysis to exclude cases where the observed absence was the result of erroneous gene-finding or incomplete annotation. We found that most VFs are present in all *P. aeruginosa* strains, even in the less virulent strains, such as the stain CF5 (8th lane of Fig. 2A and Supplementary Table 2). On the other hand, most *P. aeruginosa* VFs were absent in non-*P. aeruginosa* strains (last 12 lanes of Fig. 2A and Supplementary Table 2). Based on the VF presence-absence profile highly and lowly virulent *P. aeruginosa* strains cluster together rather than clearly apart, and separately from the other *Pseudomonas* strains (Fig. 2B). Therefore, the presence-absence differences in VFs likely reflect the differences between *P. aeruginosa* and other *Pseudomonas* strains, but not the highly from the lowly virulent *P. aeruginosa* strains. Regarding the type II secretion system (T2SS), all *P. aeruginosa* strains have all genes except from the strain PA7, which is missing the *toxA* gene, as previously published^17^. However, non-*P. aeruginosa* strains are missing almost all T2SS factors. For example, *P. putida* KT2440 is known for lacking the *toxA* gene, while the Xcp family is replaced by the Gsp family in this strain^64^.

Regarding the type III secretion system (T3SS), four *P. aeruginosa* effector proteins have been functionally identified so far, ExoU, ExoS, ExoT and ExoY^65^. In agreement with previous studies^66–71^, we observed that most of the *P. aeruginosa* strains have either the *exoS* or the *exoU* gene, but not both, while the PA7 strain carries neither of them (Supplementary Table 2). While the PA7 strain lacks the entire T3SS^17^, recent studies reveal that the cytolytic activity of PA7-like strains is based on Exolysin (ExlA), a secreted 172 kDa pore-forming toxin, exported by a two-partner secretion (TPS) system^72–74^. The *P. aeruginosa* 39016 strain is missing six T3SS genes (*exoS, exsE*, *exsC*, *pcr3*, *popD*, *pscE*), the LESB58 is missing four genes (*exoU, pcrH*, *exsE*, *pscP*) and the CF5 strain, known as a lowly virulent *P. aeruginosa* strain is only missing three genes (*exoU, exoT, pscQ*). Two T3SS genes are also missing from the strains 2192 (*exoU, exsC*) and SCV20265 (*exoU, pscE*). As with the T2SS, T3SS is nearly entirely missing from all non-*P. aeruginosa* strains, and this is in agreement with previous data on *P. putida*^64^.

Regarding the type VI secretion system, the 39016 strain is missing three genes (*hcpC*, *tagT*, *vgrG1*) and the C3719 strain is missing only one gene (*dotU*). Interestingly, *P. fluorescens* has the T6SS gene *hcp* and *P. protegens* has the *vgrG*, *hcp*, *lip*, *icmF*, *dotU*, *clpV*, *ppka* and *pppA* genes. Moreover, the *P. aeruginosa* quorum sensing genes *pzhA1*, *pzhA2*, *phzB2*, *rhlI* and *rhlR* involved in phenazine biosynthesis are missing, not only from the non-*P. aeruginosa* strains, but also from some *P. aeruginosa* strains, primarily the low in virulence strains CF5 and C3719 (Supplementary Table 2).

We proceed by clustering *Pseudomonas* strains based on the presence-absence of specific VFs for the five VF functional classes and for all VFs. The resulting clusters were further analyzed using the Adjusted Rand Index (see Materials and Methods) to reveal hidden correlations to (a) overall *Pseudomonas* phylogeny, (b) pathogenicity ranking according to the wound infection assay, and (c) pathogenicity ranking according to the oral infection assay. We found that phylogeny, but also pathogenicity to wound infection, correlate with VF gene content (Fig. 2C), indicating that VF gene content differences are due to overall *Pseudomonas* phylogeny and do not reflect directly the pathogenicity of the 30 *Pseudomonas* species. Accordingly, the phylogeny of the 30 strains correlated well with: (i) their virulence classification in the wound infection (but not in the oral infection assay); (ii) VF gene content; and (iii) major metabolic pathway gene content. To eliminate the effect of extreme phylogenetic differences due to the inclusion of non-*P. aeruginosa* strains, the analysis was repeated with only the *P. aeruginosa* strains, in which case no correlation was evident for any comparison. Thus, we reconfirm earlier works claiming that the difference in virulence potential of *P. aeruginosa* cannot be fully attributed to the mere presence or absence of known virulence factors^13^, but also to other genomic differences yet to be determined.

### Gene expression of selected VFs, but not of core metabolism genes, correlates with pathogenicity

**Figure 3:**
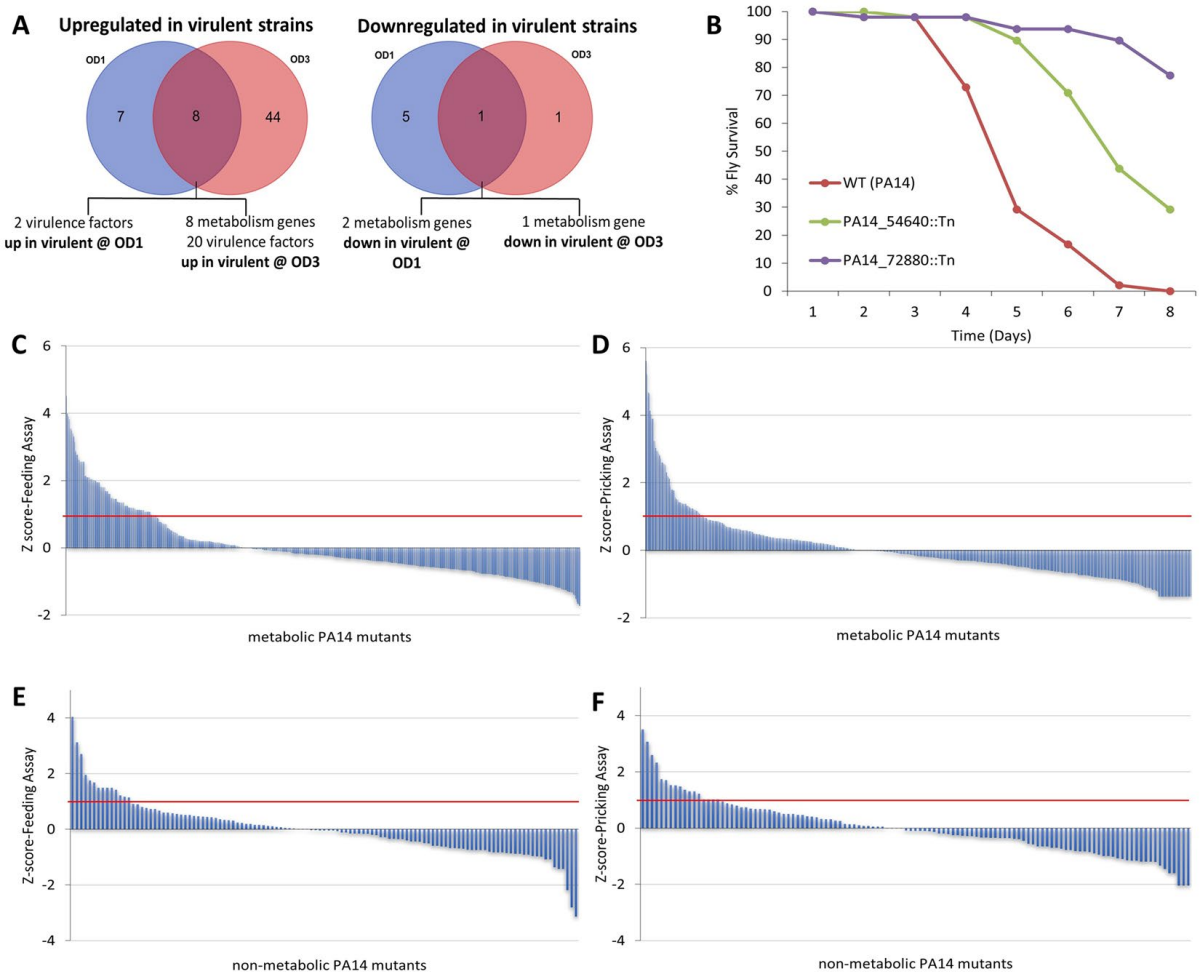
Differential strain-to-strain expression and role in virulence of *P. aeruginosa* metabolic genes. (**A,B**) Genes differentially expressed between 3 lowly and 3 highly virulent *P. aeruginosa* strains: (**A)** VENN diagrams of the 66 differentially expressed genes identified by RNAseq transcriptomics analysis using http://bioinformatics.psb.ugent.be/webtools/Venn/. At OD_600nm_: 1, 15 genes were up (VENN diagram or the left) and 6 genes were down (VENN diagram on the right) in all 3 highly vs. all 3 lowly pathogenic species. At OD_600nm_: 3, 52 were up (VENN diagram or the left) and 2 genes were down (VENN diagram or the right) in all 3 highly vs. all 3 lowly pathogenic species. (**B**) Mutants for all 10 general metabolism differentially expressed genes were tested for virulence in the fly oral infection model, and 2 of them were found attenuated. (**C–F**) Screen results for 482 metabolic and 94 non-metabolic PA14 genes using two *Drosophila* infection assays (feeding and pricking): (**C**,**D**) Z-score analysis of the fly survival after infection of flies with 553 PA14 metabolic mutants (corresponding to 482 genes) using two independent assays: Feeding Assay **(C)** and Pricking Assay **(D)**. The time of 50% fly death (LT50%) was assessed for each mutant and condition and a Z-score analysis was used to select those with a score >1 for any of the two assays, from which upon retest, 78 were found significantly attenuated in virulence per Kaplan-Meier survival analysis with a log-rank test. **(E**,**F)** Z-score analysis of the fly survival after infection of flies with 95 randomly selected non-metabolic PA14 mutants (corresponding to 94 genes) using two independent assays: Feeding Assay **(E)**, Pricking Assay **(F)**. The time of 50% fly death (LT50%) was assessed for each mutant and condition and a Z-score analysis was used to select those with a score >1 for any of the two assays, from which upon retest, 8 were found significantly attenuated in virulence per Kaplan-Meier survival analysis with a log-rank test.

To identify differentially expressed genes relating to pathogenicity we performed a transcriptome analysis of the 6 *P. aeruginosa* strains validated in mice as “high” (B136-33, MTB-1, PA14) or “low” (C3719, CF5, PACS2) in virulence (Fig. 1C). RNA was extracted from bacteria grown at mid-exponential and early stationary phase (optical density OD_600nm_: 1 and 3, respectively). We selected genes differentially regulated in the same direction (either over- or under-expressed) between all pairs of “high” vs. “low” in virulence strains. Nine genes were differentially expressed at both growth phases (8 of which were overexpressed in highly virulent strains) (Fig. 3A). At OD_600nm_: 1, 15 genes were up, and 6 genes were down in all 3 highly versus all 3 lowly pathogenic species (Fig. 3A). At OD_600nm_: 3, 52 were up and 2 genes were down in all 3 highly versus all 3 lowly pathogenic species (Fig. 3A). Overall, 20 VFs (most of them being quorum sensing and T3SS related), 10 metabolism genes, 5 transcriptional regulators and a number of hypothetical proteins were differentially expressed (Supplementary Table 5).

Among the virulence genes overexpressed in highly pathogenic species are, *exoU* and *spcU*. This is simply because they do not exist in the lowly pathogenic species assessed. Furthermore, out of the 18 *P. aeruginosa* strains, 5 (PA14, B136-33, MTB-1, NCGM2.S1 and 39016) have the *exoU* gene, and all of them, except from 39016, have next to *exoU* the *spcU* gene, a chaperone required for efficient secretion of the ExoU cytotoxin^75^. Hypothetical proteins overexpressed in highly pathogenic species include: PA14_20600, a DUF3313 domain-containing protein (PFAM ID: PF11769), a domain defining a family of putative bacterial lipoproteins; PA14_04710, a 4Fe-4S ferredoxin that, like other ferredoxins, mediates electron transfer in a wide variety of metabolic reactions^76^; PA14_30690, a fimbrial protein playing a role in cell adhesion; and PA14_01120 that maps to the antitoxin Tsi6 of the structurally characterized Tse6::Tsi6 complex, a toxin-antitoxin system and is related to T6SS^77^. Quorum sensing genes, including, the autoinducer *rhlI* and its receptor *rhlR*, the rhamnolipid biosynthesis genes *rhlA* and *rhlB*, the proteases *lasA* and *lasB*, the *pa1L* and *lecB* lectins and the phenazine biosynthesis genes *phzB1*, *phzC1*, *phzE1*, *phzM* and *phzS*, were consistently overexpressed among the 3 highly vs. the 3 lowly virulent strains (Supplementary Table 5). Thus, contrary to the inability of VF presence-absence profiles to distinguish highly from lowly pathogenic *P. aeruginosa* strains, our data indicate that overexpression of particular VFs is indicative of virulence potential. However, VF control is very complex, and additional regulating factors need to be investigated to improve our understanding on their regulation.

Among the 10 metabolic genes, 8 were found upregulated in the highly virulent strains, but mutations in none of them exhibited defects in virulence in flies. Mutations, on the other hand, in the 2 downregulated in highly virulent strains metabolic genes exhibited clear defects in virulence (Fig. 3B). Of the latter, PA14_54640 (*dspI*) encodes for a putative enoyl-CoA hydratase involved in biofilm dispersion. Its mutation produces high biofilm formation, a condition that may promote chronicity to the expense of acute virulence^78,79^. The second downregulated in highly virulent strains metabolic gene PA14_72880 encodes for the putative short-chain dehydrogenase involved in fatty acid biosynthesis. Mutations of its orthologs in PAO1 reduce biofilm formation but also swarming motility and LasB protease activity^80^. The fact that a number of metabolic genes appear among the consistently differentially expressed genes (Fig. 3A) begs the question of the broader contribution of metabolism in *P. aeruginosa* virulence^24^.

### Relative contribution of core metabolic and non-metabolic *P. aeruginosa* PA14 genes to virulence

**Figure 4:**
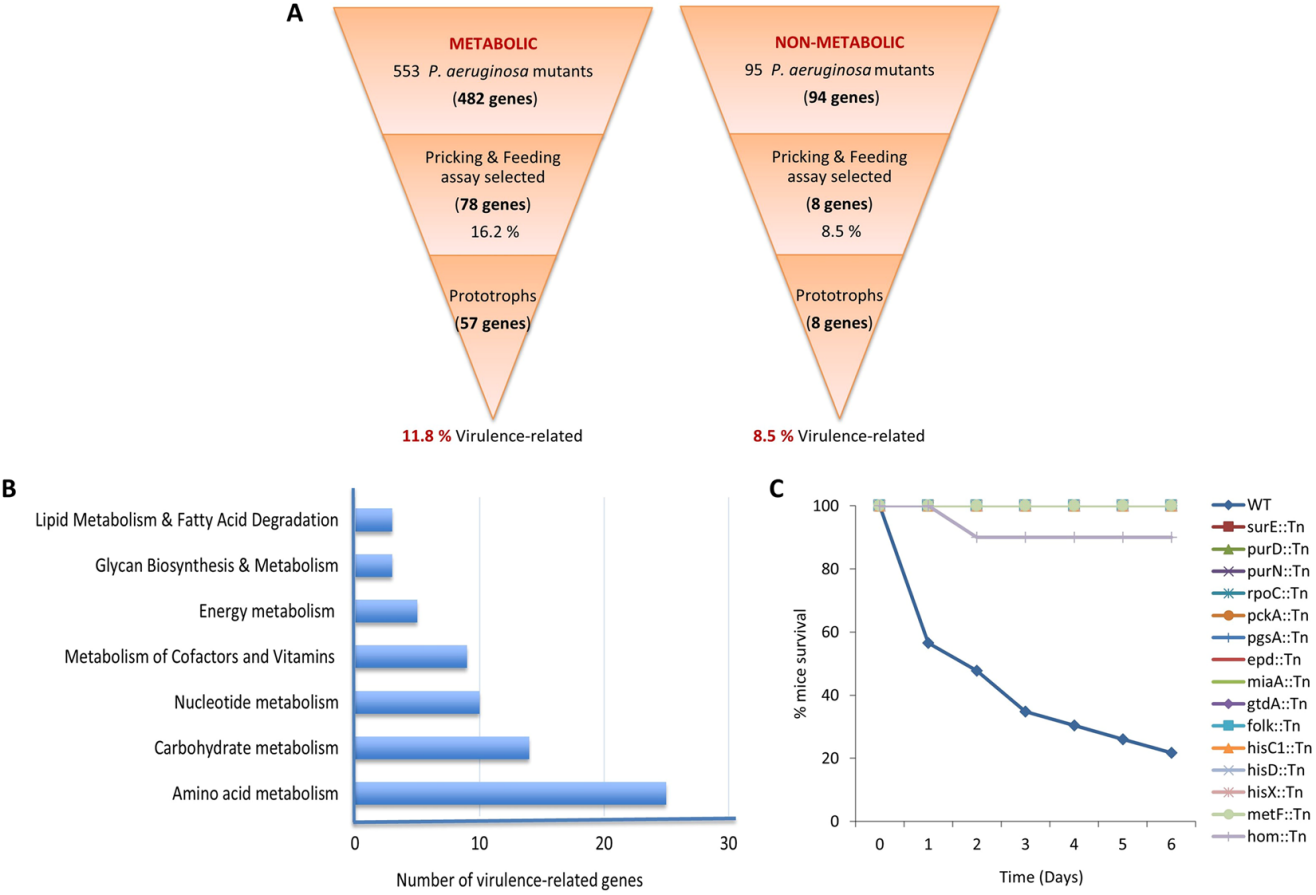
*P. aeruginosa* core metabolism genes are abundantly represented among genes required specifically for full virulence in flies and mice. (**A**) Five hundred and fifty-three (553) *P. aeruginosa* metabolic Tn-mutants corresponding to 482 core metabolism genes and 95 randomly selected non-metabolic *P. aeruginosa* Tn-mutants corresponding to 94 non-metabolic genes were screened in the wound and oral infection *Drosophila* assays. Sixteen percent (16.2%) of the core metabolism and 8.5% of the non-metabolic *P. aeruginosa* genes were selected as required for full virulence. Examination of growth capacity of the selected mutants in minimal media and in the host revealed the percentage of core metabolism genes dispensable for growth, i.e. important specifically for full virulence, is at least as high as that of the non-metabolic genes. **(B)** Assignment of the 57 PA14 virulence-related metabolic genes in metabolic categories. The 57 core metabolism *P. aeruginosa* gene mutants that grow normally in minimal media and/or in the host belong in one or more of 7 general core metabolism categories. Each category is represented by at least 3 virulence-related genes, while nucleotide, carbohydrate and amino acid metabolism categories by 10 or more. **(C)** Survival kinetics of mice after intranasal inoculation with PA14 metabolic mutants. The graph shows the % mice survival after intranasal inoculation with 15 PA14 metabolic mutants and the wild type strain. All strains, but wild type PA14 (blue) and *hom* (light purple), overlap on the horizontal 100% survival line. Twenty-microliter of saline containing 2 × 10^7^ bacteria was administered intranasally to each mouse. Infected mice were monitored for 6 days (n = 10–11).

To functionally assess the contribution of the core metabolism genes of *P. aeruginosa* to virulence, we took advantage of the PA14 unigene Transposon Insertion Mutant Library. The PA14 strain is highly pathogenic and well annotated. Its genome contains 6,537,648 nucleotides and 5,977 annotated genes^3,19^ 794 of which were annotated as metabolic in the KEGG database in the early phase of this project in 2014 (961 genes were annotated as metabolic in October 2019). We investigated the virulence in flies of all 553 available and retrievable core metabolism gene mutants of the PA14 mutant library. These correspond to 482 core metabolism genes and 95 randomly selected mutants corresponding to 94 non-metabolic genes in the pricking (wound) and feeding (oral) infection fly assays. To avoid false negatives, we manually screened 3 replicates of 10 wild type orally infected and 2 replicates of 20 wild type wound infected flies with each of the 648 PA14 mutant strains. The time of 50% fly death (LT50%) was measured for each mutant in each assay and a Z-score analysis was used to select mutants allowing fly survival to infection by more than 1 standard deviation of the normalized mean (Fig. 3C-F). To avoid false positives, selected mutants were retested for virulence and the number of the attenuated mutants was finalized after performing Kaplan-Meier survival analysis with a log-rank test. Considering both assays, 16.2% (78/482) of the metabolic and 8.5% (8/94) of the non-metabolic *P. aeruginosa* genes, were found virulence-defective in flies (Fig. 4A). Even though the number of strains tested only marginally fails to statistically support that more metabolic than non-metabolic genes are implicated in virulence (Fisher exact test: *p* = *0.0583*), the numbers clearly suggest that metabolism has a considerable contribution in *P. aeruginosa* virulence.

### Growth assessment of the virulence-defective PA14 metabolic mutants

The identified metabolism gene mutants might be impaired in growth (exhibit auxotrophy) rather than being directly involved in virulence factor production. Therefore, we sought to determine which of the 78 attenuated in virulence PA14 metabolic gene mutants are growth-essential in culture or necessary for host colonization during infection. We assessed growth in two types of minimal media (one without and one with fly extract as an extra nutrient), and the level of colonization during infection at the respective infection model (wound or oral) in which each mutant was found attenuated in virulence.

Using a standard minimal medium we initially identified 34 out of the 78 core metabolic mutants as prototrophs for being able to grow similarly to the wild type strain (Table 1). The remaining 44 core metabolic mutants did not grow at all in this medium, requiring additional nutrients for their growth. Nevertheless, if the needed nutrients are available in flies during infection, the lack of virulence should be due to a direct effect of the mutation in virulence rather than in growth. To assess this possibility, we inoculated minimal media supplemented with 5% fly extract with each of the 44 core metabolic mutants. This medium offers all the nutrients the bacterium can find in flies in the absence of the host defense against the bacteria. We found 12 of the 44 PA14 metabolic mutants to grow similarly to the wild type and considered them conditional prototrophs (Table 2 **- Group A, B**). All 8 virulence-defected non-metabolic mutant strains were also able to grow in this medium (Supplementary Fig. 2A,B) and we did not test them any further.

**Table 1:** Thirty-four virulence-related prototrophs based on growth in glucose minimal media. From the 78 virulence-defective PA14 metabolic mutants, 34 can grow like the wild type in the M9 medium, indicating a connection of the corresponding genes with virulence.

#	Metabolic PA14 mutants	*In vitro* growth	*In vivo* growth (colonization)	
		Minimal medium (M9)	Intestinal	Wound
1*	PA14_00120 (lipid A biosynthesis lauroyl acyltransferase)	+	−	−
2	PA14_04320 (*ilvA1*)	+	+	NT
3	PA14_07600 (*folk*)	+	+	NT
4*	PA14_11250 (hypothetical protein)	+	−	−
5	PA14_11810 (aldehyde dehydrogenase)	+	+	NT
6*	PA14_14680 (*suhB*)	+	−	−
7	PA14_17450 (*surE*)	+	+	NT
8*	PA14_22050 (*htrB*)	+	−	−
9	PA14_20670 (glutamine synthetase)	+	+	NT
10	PA14_29860 (*nuoM*)	+	+	NT
11	PA14_29980 (*nuoE*)	+	+	NT
12	PA14_29990 (*nuoD*)	+	+	NT
13	PA14_31580 (acyl-CoA dehydrogenase)	+	+	NT
14	PA14_32690 (*gtdA*)	+	+	NT
15	PA14_38510 (*hmgA*)	+	+	NT
16*	PA14_40980 (enoyl-CoA hydratase)	+	+	+
17	PA14_44070 (*gltA*)	+	+	NT
18	PA14_45010 (*hyi*)	+	+	NT
19	PA14_52050 (*purN*)	+	−	NT
20	PA14_52610 (hypothetical protein)	+	+	NT
21	PA14_52800 (*acsA*)	+	+	NT
22	PA14_65320 (*miaA*)	+	+	NT
23	PA14_66670 (*ponA*)	+	+	NT
24	PA14_68580 (*pckA*)	+	+	NT
25	PA14_70160 (*bioA*)	+	+	NT
26	PA14_71970 (*wbpW*)	+	+	NT
27	PA14_72850 (glutamine synthetase)	+	+	NT
28	PA14_00020 (*dnaN*)	+	NT	+
29	PA14_07170 (*epd*)	+	NT	+
30	PA14_08780 (*rpoC*)	+	NT	+
31	PA14_31700 (*pgsA*)	+	NT	+
32	PA14_32670 (hypothetical protein)	+	NT	+
33	PA14_41020 (*adi*)	+	NT	+
34	PA14_63990 (*speA*)	+	NT	+

Feeding assay-selected: 1-27; Those selected in both assays are shown with asterisk (*); Pricking assay-selected only: 28-34; NT: Not Tested.

**Table 2:** Forty-four virulence-defective mutants: 23 conditional prototrophs & 21 auxotrophs. These metabolic mutants can be grouped in metabolic pathways based on the KEGG database^25^. They are unable to grow in glucose minimal medium (M9), but 23 of them can grow “conditionally” like the wild type strain in M9 supplemented with 5% fly extract and/or in flies. The remaining 21 do not grow like the wild type in any condition tested.

#	Major Pathways (KEGG database entry)	Metabolic PA14 mutants	*In vitro* growth	*In vivo* growth (colonization)		
			Glucose minimal medium (M9) with 5% Fly Extract	Intestinal	Wound	
**1**	Histidine metabolism (pau00340)	PA14_57770 (*hisC1*)	+	+	NT	**Group (A) (# 6 mutants)** Efficient growth in glucose minimal medium with 5% fly extract and *in vivo*
**2**		PA14_57780 (*hisD*)	+	+	NT	
**3**		PA14_66950 (*hisE*)	+	+	NT	
**4**		PA14_65250 (*hisX*)	+	+	NT	
**5**	One carbon pool by folate (pau00670)	PA14_05590 (*metF*)	+	+	NT	
**6**	Pantothenate and CoA biosynthesis (pau00770)	PA14_62580 (*panB*)	+	+	NT	
**7**		PA14_62590 (*panC*)	+	−	NT	**Group (B) (# 6 mutants)** Efficient growth only in glucose minimal medium with 5% fly extract
**8**	Purine metabolism (pau00230)	PA14_51240 (*purC*)	+	−	NT	
9*		PA14_64220 (*purD*)	+	−	−	
**10**		PA14_23920 (*purF*)	+	−	NT	
11*		PA14_64200 (*purH*)	+	−	NT	
**12**		PA14_15740 (*purL*)	+	−	NT	
13*	Pyrimidine metabolism (pau00240)	PA14_05260 (*pyrB*)	−	+	−	**Group (C) (# 11 mutants)** Efficient growth only *in vivo*
14*		PA14_05250 (*pyrC*)	−	−	+	
15*	Glycine, serine and threonine metabolism (pau00260)	PA14_16070 (*hom*)	−	+	+	
16*		PA14_16090 (*thrC*)	−	−	+	
**17**	Arginine biosynthesis (pau00220)	PA14_08480 (*argC*)	−	+	NT	
18*		PA14_18740 (*argG*)	−	+	NT	
**19**		PA14_18610 (*argF*)	−	NT	+	
**20**	Glycolysis/Gluconeogenesis (pau00010)	PA14_66310 (*aceF*)	−	+	NT	
21*		PA14_66290 (*aceE*)	−	+	NT	
**22**	Cysteine and methionine metabolism (pau00270)	PA14_05620 (*sahH*)	−	−	+	
**23**	Lysine biosynthesis (pau00300)	PA14_69670 (*lysA*)	−	+	NT	
**24**	Phenylalanine, tyrosine and tryptophan biosynthesis (pau00400)	PA14_00450 (*trpB*)	**Group (D) (# 21 mutants)** Slow growth compared to the wild type PA14 in all the above assays			
**25**		PA14_08360 (*trpC*)				
**26**		PA14_08350 (*trpD*)				
**27**		PA14_23850 (*trpF*)				
**28**		PA14_66600 (*aroB*)				
**29**	Valine, leucine and isoleucine biosynthesis (pau00290)	PA14_62130 (*ilvC*)				
**30**		PA14_04630 (*ilvD*)				
**31**		PA14_62150 (*ilvH*)				
**32**		PA14_62160 (*ilvI*)				
**33**		PA14_23790 (*leuB*)				
**34**		PA14_23750 (*leuC*)				
35*	Pyrimidine metabolism (pau00240)	PA14_18710 (*pyrC*)				
36*		PA14_24640 (*pyrD*)				
**37**		PA14_26890 (*pyrF*)				
38*		PA14_62930 (*carA*)				
**39**	Pentose phosphate pathway (pau00030)	PA14_22910 (*edd*)				
**40**		PA14_23090 (*edaA*)				
**41**	Arginine biosynthesis (pau00220)	PA14_70280 (*argB*)				
**42**		PA14_69500 (*argH*)				
**43**	Glycine, serine and threonine metabolism (pau00260)	PA14_65560 (*serB*)				
**44**	Glycolysis/Gluconeogenesis (pau00010)	PA14_62830 (*tpiA*)				

*: Found attenuated in both assays (pricking and feeding); NT: Not Tested.

Moreover, we sought to examine the 78 metabolic mutant strains during initial colonization, to identify auxotrophic strains able to colonize the flies, but also to study the colonization ability of the prototrophs. We adapted our wound and intestinal infection assays to assess colonization efficiency rather than fly survival. To assess intestinal colonization we infected flies for only one day with all flies being subsequently transferred to tubes with clean food, every day for 3 days. To assess wound colonization we injected the bacteria in the flies instead of pricking them with a tungsten needle to bypass the fly immune system that can easily eliminate attenuated in virulence bacteria at the wound site^30^. Calculating the number of retrievable bacteria per fly we found that 29 of the 34 prototrophs (Table 1) and 17 of the remaining 44 strains (Table 2 **- Group A, C**) were able to colonize the flies like the wild type. In summary, we found 34 prototrophs (Table 1), 23 conditional prototrophs able to grow either in the host or in minimal medium cultures supplemented with fly extract (Table 2**, Group A-C**) and 21 auxotrophs unable to grow in the host or in culture (Table 2**, Group D**). Accordingly, 11.8% (57/482) of the core metabolic and 8.5% (8/94) of the non-metabolic attenuated strains were categorized as virulence-related (Fig. 4A), suggesting that many and functionally disparate metabolism genes are connected to *P. aeruginosa* virulence.

### Virulence-related metabolic genes of *P. aeruginosa* PA14 are part of core metabolism and are necessary for full virulence in mice

According to the KEGG database, the selected 57 virulence-related core metabolic genes belong to 7 metabolic categories and various metabolic gene pathways (Fig. 4B). Assessing the virulence in a mouse lung infection assay of mutants representing each core metabolic pathway as defined in the KEGG database we identified 15 mutants being virulence-defective in flies and mice. These mutants affect genes related to amino acid metabolism [PA14_57770 (*hisC1*), PA14_57780 (*hisD*), PA14_65250 (*hisX*), PA14_32690 (*gtdA*), PA14_16070 (*hom*)], nucleotide metabolism [PA14_17450 (*surE*), PA14_64220 (*purD*), PA14_52050 (*purN*)] and the metabolism of co-factors and vitamins [PA14_05590 (*metF*), PA14_07170 (*epd*), PA14_07600 (*folKfolk*)] (Fig. 4C and Table 3).

**Table 3:** Pathways of the 15 PA14 metabolic genes corresponding to mutants found attenuated in mice lung infection. The names of the metabolic genes are listed in the first column. The second column shows the corresponding metabolic pathways based on the KEGG database^25^, while the last column shows the major central metabolic pathways in which those pathways belong.

#	Genes/Names	Metabolic Pathways (KEGG database entry)	Major Central Metabolic Pathways
**1**	PA14_17450 (*surE*)	Purine (pau00230), Pyrimidine (pau00240) & Nicotinate and nicotinamide metabolism (pau00760)	Nucleotide metabolism Metabolism of cofactors and vitamins
**2**	PA14_64220 (*purD*)	Purine metabolism (pau00230)	
**3**	PA14_52050 (*purN*)	Purine metabolism (pau00230), One carbon pool by folate (pau00670)	
**4**	PA14_08780 (*rpoC*)	RNA polymerase (pau03020)	
**5**	PA14_57770 (*hisC1*)	Histidine metabolism (pau00340) & Phenylalanine, tyrosine and tryptophan biosynthesis (pau00400)	Amino acid metabolism
**6**	PA14_57780 (*hisD*)	Histidine metabolism (pau00340)	
**7**	PA14_65250 (*hisX*)	Histidine metabolism (pau00340)	
**8**	PA14_32690 (*gtdA*)	Tyrosine metabolism (pau00340)	
**9**	PA14_16070 (*hom*)	Glycine, serine and threonine metabolism (pau00260), Cysteine and methionine metabolism (pau00270) & Lysine biosynthesis (pau00300)	
**10**	PA14_05590 (*metF*)	One carbon pool by folate (pau00670)	Metabolism of cofactors and vitamins
**11**	PA14_07170 (*epd*)	Vitamin B6 metabolism (pau00750)	
**12**	PA14_07600 (*folk*)	Folate Biosynthesis (pau00790)	
**13**	PA14_68580 (*pckA*)	Glycolysis/Gluconeogenesis (pau00010), TCA cycle (pau00020), Pyruvate metabolism (pau00620)	Carbohydrate metabolism
**14**	PA14_65320 (*miaA*)	tRNA dimethylallyltransferase (pau01100)	Transfer RNA biogenesis
**15**	PA14_31700 (*pgsA*)	Glycerophospholipid metabolism (pau00564)	Lipid metabolism

### Virulence-related metabolic genes of *P. aeruginosa* PA14 are compromised in various aspects of virulence

**Figure 5:**
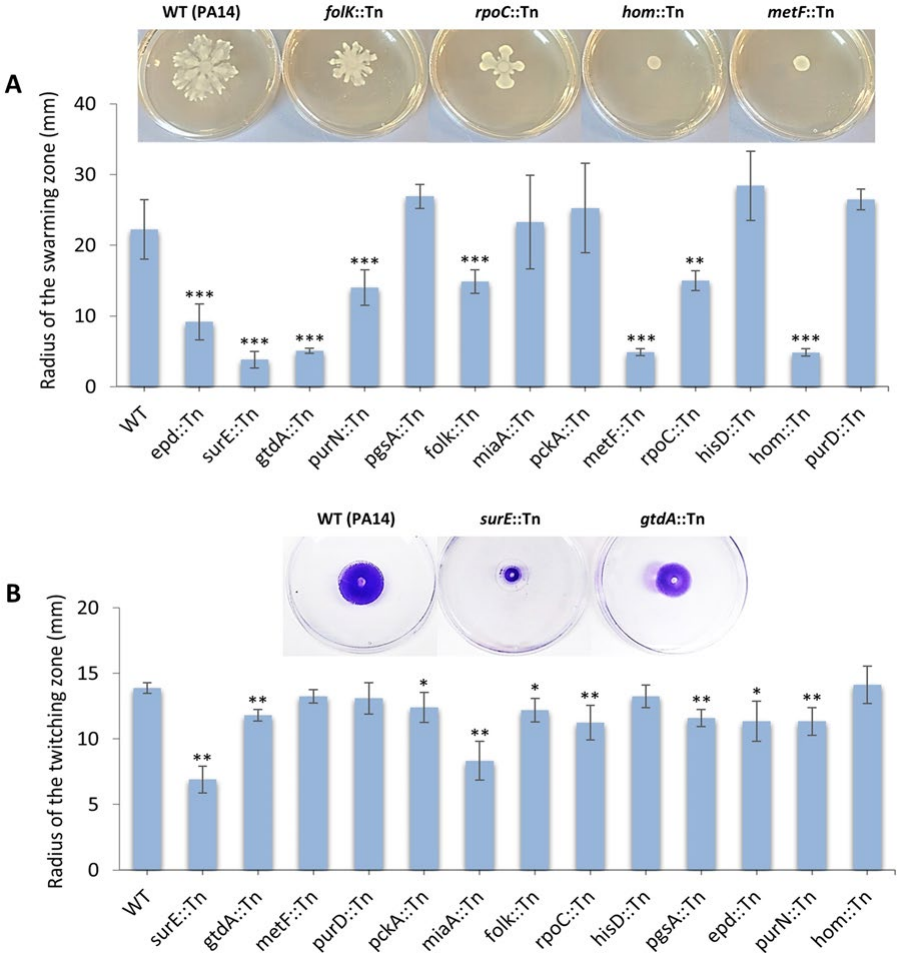
Swarming and twitching phenotypes of 13 PA14 metabolic gene mutants. (**A**) Swarming motility was measured by the length of the swarming zone from the center of the plate after inoculation of the cells at the center of the agar media and incubation at 37 °C for 24 h. The results are from 3 independent experiments. n = 3-13; **P < 0.01; ***P < 0.001 compared to WT by Mann-Whitney *U* test. Representative photos of Petri dishes exhibiting wild type PA14 and selected mutants with defective swarming motility, shown above the graph. **(B)** For the twitching motility the cells were stab-inoculated onto LB twitching plates (1% agar). The plates were incubated at 37 °C for 48 hours. The agar was removed, and the twitching zone was revealed by staining with crystal violet, measuring the maximum length of the twitching zone from the center of the plate. The results are from 3 independent experiments. n = 4-7; *P < 0.05; **P < 0.01 compared to WT by Mann-Whitney *U* test. Representative photos of Petri dishes exhibiting wild type PA14 and selected mutants with defective twitching motility, shown above the graph. In both (A) and (B) error bars depict standard deviation.

To better understand the loss in virulence of the selected metabolic gene mutants, we studied them for the production of virulence factors associated with acute infection, namely, bacterial motility, and T3SS and quorum sensing gene expression. Swarming and twitching motility is very important for *P. aeruginosa* virulence^81^ and we tested both types of motility of 13 PA14 metabolic transposon (Tn) mutants found attenuated in both flies and mice. Mutants for *epd*, *surE*, *gtdA*, *purN*, *folK*, *metF*, *hom* and *rpoC*, were noticeably defective for swarming motility compared to the wild type strain (*P* < 0.001 for the first 7 and *P* < 0.01 for the last one), while *pgsA* and *purD*, exhibited increased swarming motility compared to the wild type (*P* < 0.05) (Fig. 5A). Regarding the twitching motility, the mutants *surE*, *gtdA*, *miaA*, *rpoC, pgsA* and *purN* exhibited a strong decrease in their ability to twitch compared to the wild type (*P* < 0.01) (Fig. 5B), but also the *pckA*, *folK* and *epd* mutants were significantly defective in twitching motility (*P* < 0.05) (Fig. 5B). Only *hisD* and *purD* Tn-mutants were not impaired in any of the two motilities.

**Figure 6:**
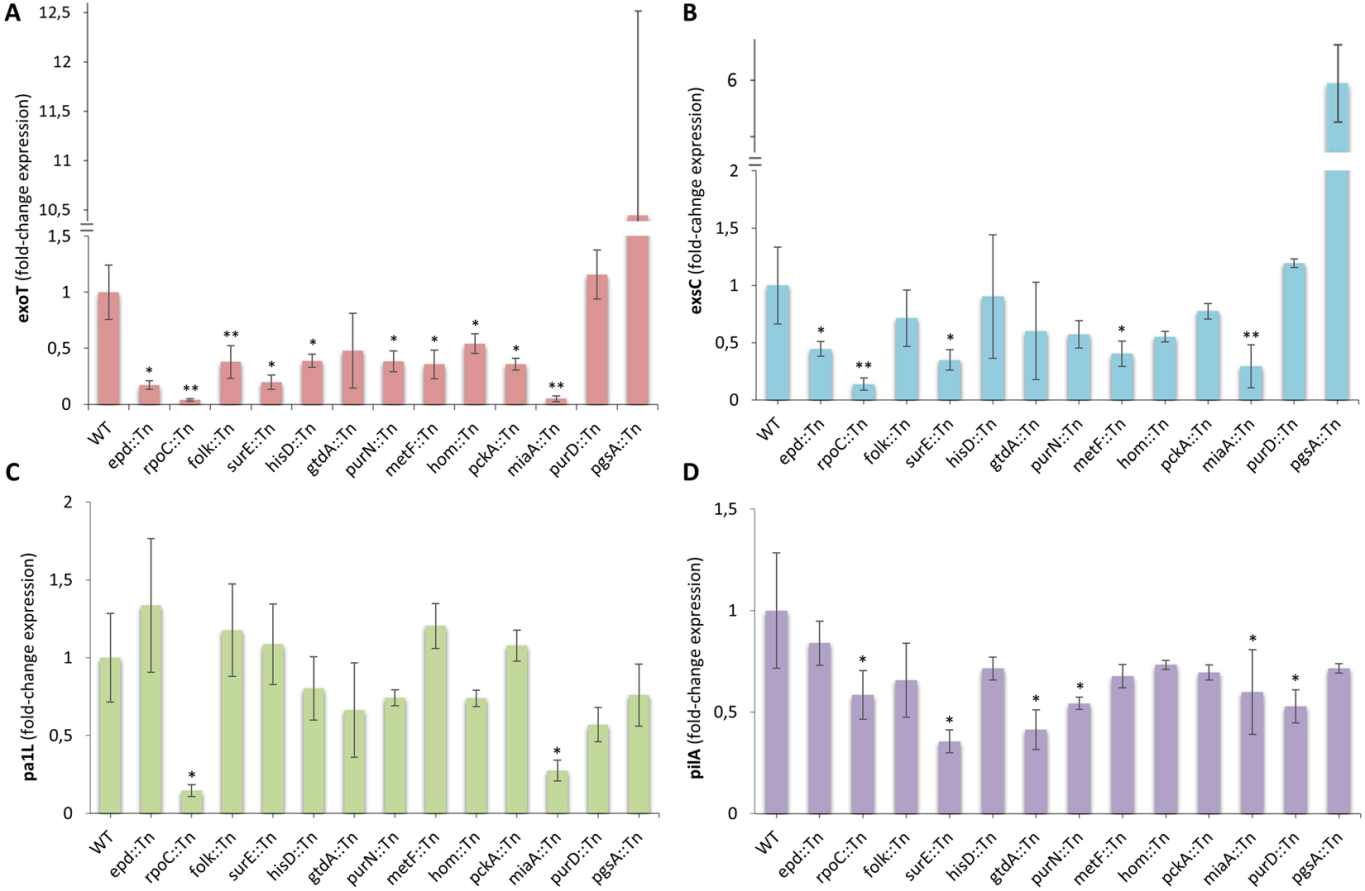
Expression of various virulence factors of 13 PA14 metabolic gene mutants. (**A,B**) Expression of T3SS genes in PA14 metabolic mutants. **(C,D)** Expression of *pa1L* and type IV pilus gene *pilA* in PA14 metabolic mutants. Bacteria were grown to an OD_600nm_ of 1 in LB with 5 mM EGTA before RNA extraction and cDNA synthesis. *P < 0.05; **P < 0.01 compared to WT by Mann-Whitney *U* test (n = 3–7). In all plots (**A**–**D**) the average fold change ± standard deviation is shown.

Gram-negative bacterial pathogens have evolved multiple protein secretion systems that facilitate the infection of eukaryotic hosts. *P. aeruginosa* deploys its type III secretion system (T3SS) to enhance its pathogenicity by injecting cytotoxic effector proteins into the host cells^82^. T3SS expression is regulated transcriptionally and post-transcriptionally in response to host cell contact and environmental Ca^2+^ levels^83^. We examined the expression of the T3SS regulatory gene *exsC* and the effector protein *exoT*, in the 13 Tn-mutants, under Ca^2+^ limiting conditions (LB supplemented with 5 mM EGTA). We observed reduced expression of the *exoT* in 10 of the 13 selected mutants and overexpression in 1 of them, compared to the wild type (Fig. 6A). Similarly, the expression of *exsC* is reduced in 5 of these mutants, with only *purD* remaining unaffected in the expression of any of the two genes (Fig. 6B). Nevertheless, *purD* as well as the *miaA*, *rpoC*, *surE*, *purN* and *gtdA* mutants were compromised in the expression of the type IV pilus biogenesis gene *pilA* (Fig. 6D), while the *pa1L* gene, which is controlled by quorum sensing is significantly affected by *miaA, rpoC* and tentatively by *purD* (Fig. 6C). *miaA*, is a tRNA isopentenyl transferase, a tRNA modification enzyme important for translation efficiency, while *rpoC* is the DNA-directed RNA polymerase beta subunit that has an important role in transcription. Considering that total disruption of *rpoC* might be lethal for *P. aeruginosa*^84^, the transposon mutant available for *rpoC* in the library we used might be a hypomorph with reduced gene expression or protein function due to the transposon insertion being at the end of the gene. Assuming off-target effects of transposon mutagenesis are absent from most of the mutants, our results suggest that loss of metabolic gene function impacts acute virulence by compromising one or more key mechanisms of *P. aeruginosa* virulence.

### Core metabolism pathways containing necessary for full virulence and virulence-related differentially expressed genes

To pinpoint metabolic gene expression patterns important for *P. aeruginosa* virulence, we assessed the differential expression of the 78 core metabolism genes required for the full virulence of *P. aeruginosa* strain PA14. Comparisons were performed for the 3 selected as high vs. the 3 selected as low in virulence *P. aeruginosa* strains for each of the bacterial growth phases. 45 of the 78 functionally important metabolic genes did not exhibit differential expression among any of the 18 comparisons performed. Of the remaining 33 genes, 32 exhibited a significant differential expression in up to 8 of the total 18 pairwise combinations (Supplementary Fig. 3). Thus, no consistent differential gene expression is noted for any but one of the 78 core metabolism genes required for full virulence.

**Figure 7:**
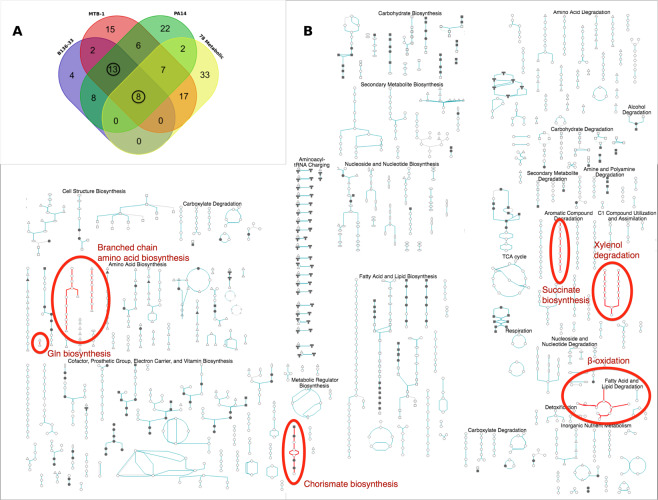
(**A**) Overlap among 4 metabolic gene pathway groups. Three expression-based metabolic pathway groups arise from differentially expressed genes comparing each of the 3 high (B136–33, MTB-1 or PA14) with all 3 low (CF5, C3719 and PCS2) in virulence strains, while the 4th functionality-based metabolic pathway group arises from the 78 genes functionally important for full virulence in flies. Out of 137 metabolic pathways assessed for overlap based on the BioCyc Database Collection (https://biocyc.org), 21 were common to the 3 expression-based metabolic pathway groups, while 8 of them were also functionally important for virulence. We circle the numbers of the 8 functionality-based plus 13 more, totaling 21 common expression-based metabolic pathways. **(B)** Common metabolic pathways implicated in *P. aeruginosa* virulence. Highlighted are the common pathways shown in **(A)** within the Cellular Overview of *P. Aeruginosa* PA14 metabolism from BioCyc.

However, using the BioCyc Pathway/Genome Database Collection we identified 107 differentially expressed metabolic pathways containing genes differentially expressed between at least one highly (PA14, MTB-1, B136-33) vs. all 3 lowly (C3719, CF5, PACS2) virulent strains and pathways containing one or more of the 78 genes necessary for full virulence. Overlapping these pathways, we find 8 pathways containing genes upregulated in all 3 highly virulent strains vs. all 3 lowly in virulence strains and at least one gene required for full virulence when mutated (Fig. 7A**;** Supplementary Table 6). These common pathways are annotated as beta-oxidation, xylenol degradation, Gln biosynthesis, branched chain amino acid biosynthesis, succinate biosynthesis, citramalate biosynthesis, and chorismate biosynthesis (Fig. 7B); and their functions include: (i) the 4-hydroxyl-phenylacetate degradation and succinate production, (ii) the glutamine biosynthesis from glutamic acid, (iii) the shikimate and chorismate biosynthesis from D-erythrose 4-phosphate, (iv) the branched-chain amino acid biosynthesis of leucine, (v) the 2,5- and 3,5-xylenol degradation to citramalate, and (vi) the beta-oxidation of fatty acids. Of note, genes required for full virulence when mutated and those upregulated in each of the highly virulent strains are not usually the same, but they frequently belong in the same pathway (Supplementary Fig. 4). Therefore, differences in virulence among *P. aeruginosa* strains maybe partly due to the differential expression of genes belonging in these core metabolism pathways.

## Discussion

Aspects of bacterial metabolism have been linked to virulence, including the T3SS^85^, the regulation of exotoxin A expression^86,87^ and the adaptation to the host nutritional environment^88^. Carbon source availability, for example, affects virulence genes presumably as an adjustment to the host micro-environment^89^. *P. aeruginosa* gene AaaA is an arginine-specific autotransporter providing a fitness advantage in chronic wound infections where the sole source of nitrogen is peptides with an aminoterminal arginine^90^. In addition, *P. aeruginosa* CbrA is a sensor kinase involved in carbon and nitrogen utilization, which affects swarming, biofilm formation, cytotoxicity, and antibiotic resistance^91^. Computational screening further elucidates interconnectivity between *P. aeruginosa* virulence factor synthesis and growth with a metabolic network as a denominator^24^. Such studies support the notion that core metabolism genes are growth-essential and unsuitable drug targets due to their high potential for resistance development^24^. Our functional transcriptomics approach though suggests the existence of a widespread growth-independent effect of core metabolism gene expression in virulence.

We defined as virulence genes those necessary to the full virulence of *P. aeruginosa* independently of their effect on bacterial growth or host colonization ability. Thus, we included as growth-independent, mutants able to grow similarly to the wild type strain in minimal media (M9) or M9 supplemented with fly extract. We also included attenuated in virulence mutants able to colonize the host as efficiently as the wild type isogenic strain PA14. We found that 11.8% of the growth-independent core metabolism genes and 8.5% of the non-metabolic genes are directly linked to virulence. This is in agreement with previous studies linking primary with secondary virulence-related metabolites for being synthesized using the same precursor molecules^92^. For example, chorismate is the last common precursor for the biosynthesis of the primary aromatic metabolites phenylalanine, tyrosine and tryptophan as well as of the secondary aromatic metabolites phenazine-1-carboxylic acid and pyocyanin, which are known virulence factors^92^. *P. aeruginosa* utilizes the siderophore pyochelin during iron limitation, which is synthesized by the conversion of chorismate to isochorismate by the isochorismate synthase PchA and of isochorismate to salicylate by the isochorismate-pyruvate lyase PchB^93^. In addition, the primary metabolite anthranilate is a precursor for the *P. aeruginosa* quinolone signal (PQS), which is a secondary metabolite having a crucial role in quorum sensing^94^. Of note, the other major siderophore produced by *P. aeruginosa*, pyoverdine, is synthesized from L-ornithine-derived hydroxamates and L-lysine biosynthesis intermediate products^95^.

In this study, histidine metabolism mutants were considered conditional prototrophs for being able to grow efficiently in minimal media supplemented with fly extract and to colonize the flies as efficiently as the wild type *P. aeruginosa* strain. The virulence attenuation of these mutants is more likely due to defects in virulence rather than in growth. Similarly, most of the purine metabolism mutants were considered conditional prototrophs for being able to grow in minimal media that contained fly extract, but these mutants were unable to colonize flies to the same extent as the wild type strain. This suggests that although there are enough nutrients to sustain growth in the host tissues, bacteria are unable to acquire them through their virulence factors. Interestingly, we also identified conditional prototrophs that although unable to grow efficiently in culture in any of the two types of minimal media, they could colonize flies normally, thus the corresponding genes may help to damage the host, irrespective of their contribution to growth.

To validate our approach, we assessed VF production in a subset of the metabolic gene mutants that were attenuated in virulence not only in flies but also in a mouse lung infection model. We found that all of them exhibited defects in at least one aspect of virulence and most of them are involved in metabolic steps absent from the human metabolism network. Thus, many metabolic genes could be targets for anti-infective therapies against acute *P. aeruginosa* virulence. It is nevertheless questionable whether prioritization in pharmacological targeting should be given to virulence-related metabolic genes also affecting growth or those that do not affect growth.

Moreover, we found differential expression of many known virulence factors and some metabolism genes through classical transcriptome analysis of highly vs. lowly pathogenic *P. aeruginosa* strains. Combinatorial functional transcriptomics analysis at the pathway level was much more informative, revealing metabolic pathways containing differentially expressed genes between all 3 highly virulent vs. all 3 lowly virulent strains and genes required for full virulence: (i) Aromatic compound degradation/biosynthesis, including the 4-hydroxyl-phenylacetate degradation for succinate production (Succinate biosynthesis), the 2,5- and 3,5-xylenol degradation to citramalate (Xylenol degradation) and shikimate and chorismate biosynthesis from D-erythrose 4-phosphate (Chorismate biosynthesis), (ii) Amino acid biosynthesis, including branched chain amino acid biosynthesis and the glutamine biosynthesis from glutamic acid (Gln biosynthesis), and (iii) Fatty acid and lipid degradation (beta-oxidation). Thus, *P. aeruginosa* virulence can be analyzed at the transcriptome and functional level using common core metabolism modules that control and indicate the virulence of disparate *P. aeruginosa* strains.

While the impact of specific core metabolism genes is not discernible at the genomic level, interesting patterns emerge. By classifying the 30 fully sequenced *Pseudomonas* species experimentally in two different *Drosophila* infection assays (wound and oral infection) as low, medium or high in virulence, we found that the phylogeny of the 30 strains correlates well with their virulence classification in the wound infection, but not in the oral infection assay. This is probably because the wound infection is more acute and guided by highly efficient virulence-related genes, as opposed to the intestinal environment in which *Pseudomonas* is less virulent. The phylogeny of the 30 strains also correlates well with virulence factor gene content, which is expected since *P. aeruginosa* species have their own repertoire and group together in the phylogenetic tree and separately from the non-*P. aeruginosa* strains. Similarly, the phylogeny of the 30 strains also correlates with major metabolic pathway gene content. Thus, the existing correlations between pathogenicity of the 30 *Pseudomonas* strains and gene content cannot be attributed specifically to VFs or metabolic genes. The gene content of the 30 *Pseudomonas* spp. regarding five metabolic pathways (carbon, glycolysis, pyrimidine, propanoate metabolism and valine, leucine, isoleucine degradation) explains their virulence classification upon the wound infection. Nevertheless, when the analysis is repeated with only the *P. aeruginosa* strains, no correlation is evident for any comparison. Thus, the existing correlation between the pathogenicity of the 30 *Pseudomonas* strains and genomic content in metabolic genes cannot be clearly attributed to specific genes, but rather to broader interspecies differences. Given the difference in expression of known virulence factors and core metabolism pathways in distinguishing highly from lowly virulent strains, it is likely that gene promoter differences in addition to the presence of VFs and core metabolism genes may account for the total strain-to-strain variation in virulence potential.

## Supplementary information

Supplementary Information.

Supplementary Information2.

## Data Availability

All data generated or analysed during this study are included in this published article (and its Supplementary Information files), expect from the RNA-Seq FASTQ files, which are publicly accessible through the Gene Expression Omnibus database, GEO Series accession number GSE142464 and the following link: https://www.ncbi.nlm.nih.gov/geo/query/acc.cgi?acc=GSE142464 Materials and protocols generated during the current study are available from the corresponding authors on reasonable request.
