# Supplementary material for: Pseudomonas aeruginosa core metabolism exerts a widespread growth-independent control on virulence
Source: Sci Rep. 2020 Jun 11;10:9505. doi: 10.1038/s41598-020-66194-4 (PMC7289854; doi:10.1038/s41598-020-66194-4)
Supplement: Supplementary file 2 — Supplementary Information2. [file 41598_2020_66194_MOESM2_ESM.pdf]

Supplementary Figures

Tittle: *Pseudomonas aeruginosa* core metabolism exerts a widespread growth-independent control on virulence

Authors:

Stavria Panayidou[1], Kaliopi Georgiades[1,2#], Theodoulakis Christofi[1#], Stella Tamana[2#], Vasilis Promponas[2*] and Yiorgos Apidianakis[1*]

[1]Infection and Cancer Laboratory, Department of Biological Sciences, University of Cyprus, Nicosia, Cyprus
[2]Bioinformatics Research Laboratory, Department of Biological Sciences, University of Cyprus, Nicosia, Cyprus
# These authors contributed equally to this work
*Corresponding authors VP: vprobon@ucy.ac.cy and YA: apidiana@ucy.ac.cy

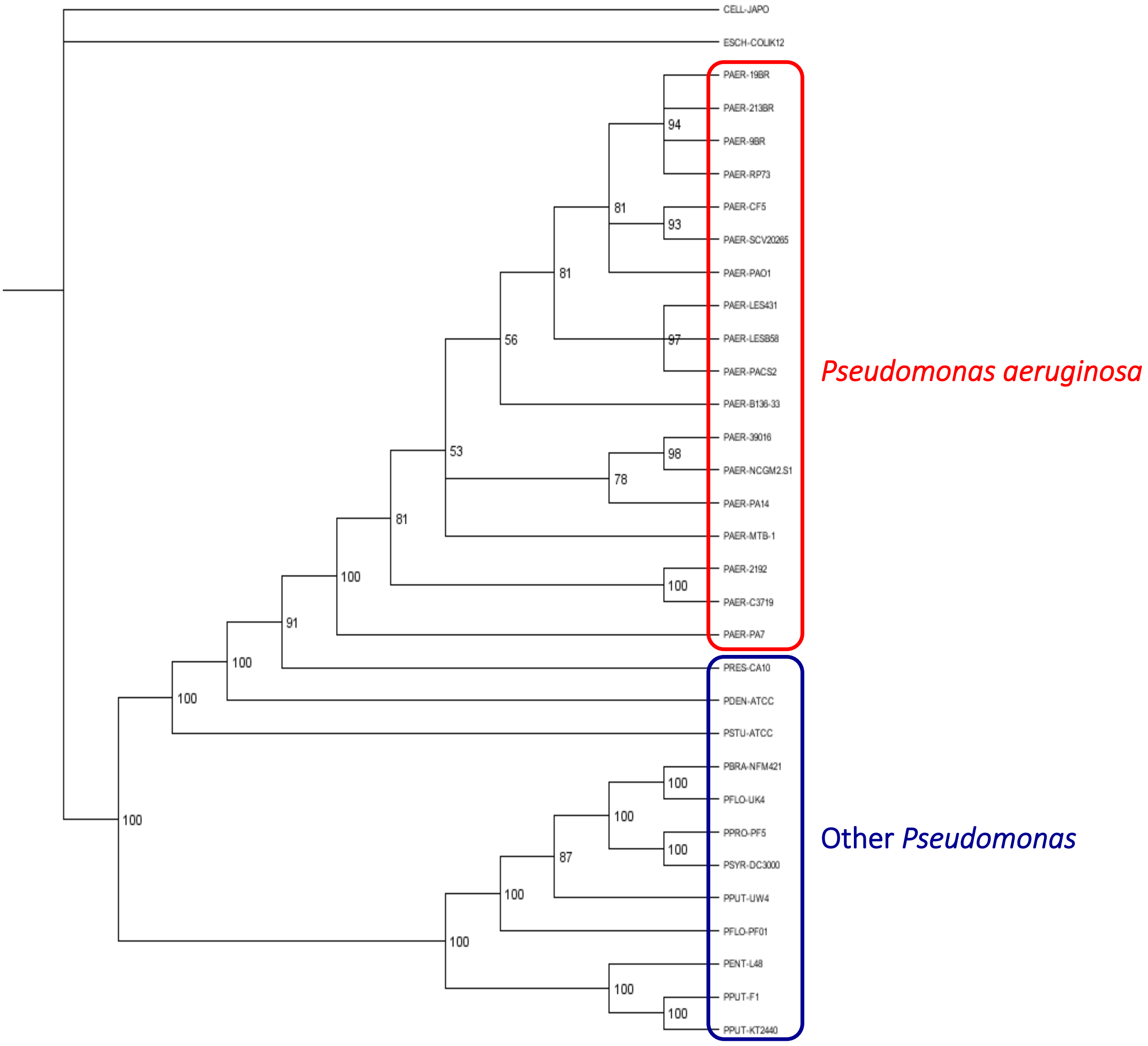

Suppl. Figure 1. Phylogenetic tree of the 18 *P. aeruginosa* and the 12 other *Pseudomonas* strains using the Bayesian Model and the 16S rRNA and gyrB gene sequences.

(per MrBayes, http://brahms.biology.rochester.edu/software.html, 2001)

A

Non-metabolic mutants		
Attenuated in the Pricking Assay	Attenuated in the Feeding Assay	Attenuated in both assays
PA14_20730	PA14_10370	PA14_25110
PA14_27280	PA14_23990	
PA14_48830	PA14_70390	
PA14_58550		

B

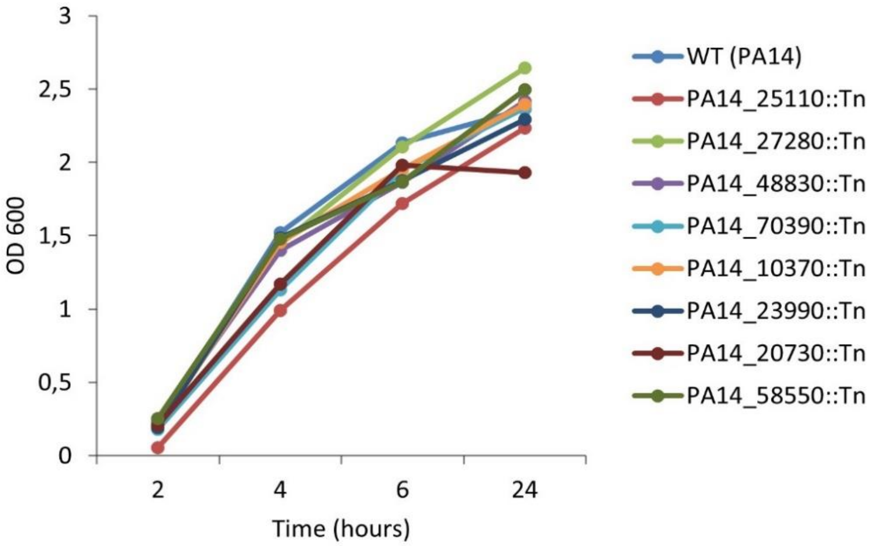

Suppl. Figure 2. Selected non-metabolic PA14 mutants and assessment of their growth in glucose minimal media supplemented with 5% fly extract.

(**A**) Non-metabolic PA14 transposon mutants that found attenuated in flies during wound and/or oral infection. The table shows all the virulence-related non-metabolic mutants and the assay in which were found attenuated.

(**B**) Growth of selected non-metabolic PA14 mutants in glucose minimal media supplemented with 5% fly extract. The growth of the selected non-metabolic mutants was assessed only in glucose minimal medium that additionally contained 5% fly extract to verify that these mutants have the potential to grow in flies. The optical density was measured at four time points. All of them were able to grow in this medium at the same extent as the wild-type PA14.

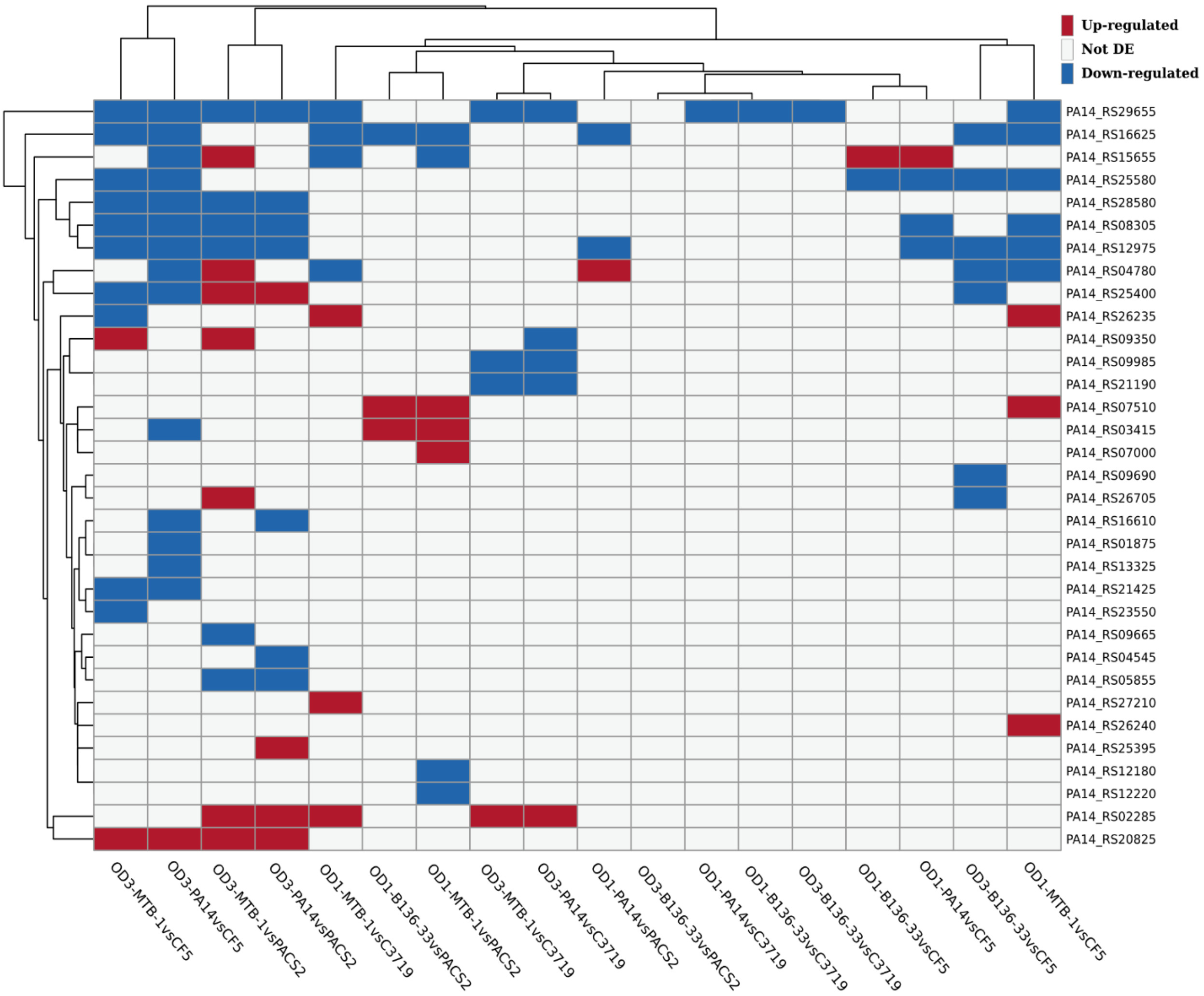

Suppl. Figure 3. Differential gene expression of core metabolism genes compared between 18 different high vs. low in virulence conditions. Display of the 33 genes significantly up- (red boxes) or down-regulated (blue boxes) in at least one of the 18 comparisons performed. Thirty-two of the 33 genes, exhibit altered expression in only 8 or less of these comparisons. No centering or scaling was applied to rows and columns of the data matrix. Both rows and columns were clustered using Manhattan distance and average linkage hierarchical clustering.

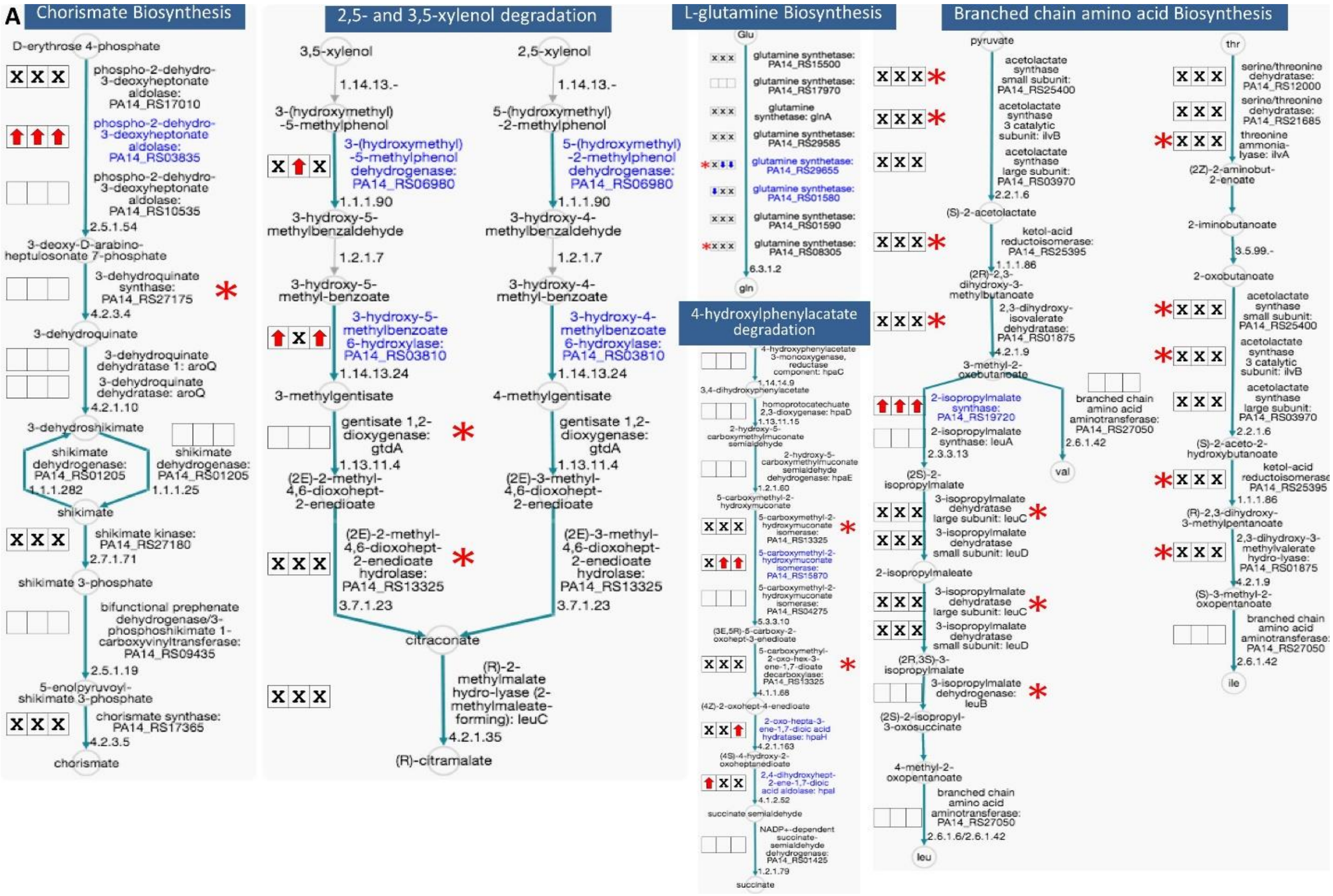
A
Chorismate Biosynthesis
D-erythrose 4-phosphate
phospho-2-dehydro-3-deoxyheptonate aldolase: PA14_RS17010
phospho-2-dehydro-3-deoxyheptonate aldolase: PA14_RS03835
phospho-2-dehydro-3-deoxyheptonate aldolase: PA14_RS10535
2.5.1.54
3-deoxy-D-arabino-heptulosonate 7-phosphate
3-dehydroquinate synthase: PA14_RS27175
4.2.3.4
3-dehydroquinate
3-dehydroquinate dehydratase 1: aroQ
3-dehydroquinate dehydratase: aroQ
4.2.1.10
3-dehydroshikimate
shikimate dehydrogenase: PA14_RS01205
1.1.1.282
shikimate dehydrogenase: PA14_RS01205
1.1.1.25
shikimate
shikimate kinase: PA14_RS27180
2.7.1.71
shikimate 3-phosphate
bifunctional prephenate dehydrogenase/3-phosphoshikimate 1-carboxyvinyltransferase: PA14_RS09435
2.5.1.19
5-enolpyruvoyl-shikimate 3-phosphate
chorismate synthase: PA14_RS17365
4.2.3.5
chorismate
2,5- and 3,5-xylenol degradation
3,5-xylenol
1.14.13.-
3-(hydroxymethyl)-5-methylphenol
3-(hydroxymethyl)-5-methylphenol dehydrogenase: PA14_RS06980
1.1.1.90
3-hydroxy-5-methylbenzaldehyde
1.2.1.7
3-hydroxy-5-methyl-benzoate
3-hydroxy-5-methylbenzoate 6-hydroxylase: PA14_RS03810
1.14.13.24
3-methylgentisate
gentisate 1,2-dioxygenase: gtdA
1.13.11.4
(2E)-2-methyl-4,6-dioxohept-2-enedioate
(2E)-2-methyl-4,6-dioxohept-2-enedioate hydrolase: PA14_RS13325
3.7.1.23
2,5-xylenol
1.14.13.-
5-(hydroxymethyl)-2-methylphenol
5-(hydroxymethyl)-2-methylphenol dehydrogenase: PA14_RS06980
1.1.1.90
3-hydroxy-4-methylbenzaldehyde
1.2.1.7
3-hydroxy-4-methyl-benzoate
3-hydroxy-4-methylbenzoate 6-hydroxylase: PA14_RS03810
1.14.13.24
4-methylgentisate
gentisate 1,2-dioxygenase: gtdA
1.13.11.4
(2E)-3-methyl-4,6-dioxohept-2-enedioate
(2E)-3-methyl-4,6-dioxohept-2-enedioate hydrolase: PA14_RS13325
3.7.1.23
citraconate
(R)-2-methylmalate hydro-lyase (2-methylmaleate-forming): leuC
4.2.1.35
(R)-citramalate
L-glutamine Biosynthesis
Glu
glutamine synthetase: PA14_RS15500
glutamine synthetase: PA14_RS17970
glutamine synthetase: glnA
glutamine synthetase: PA14_RS29585
glutamine synthetase: PA14_RS29655
glutamine synthetase: PA14_RS01580
glutamine synthetase: PA14_RS01590
glutamine synthetase: PA14_RS08305
6.3.1.2
gln
4-hydroxylphenylacatate degradation
4-hydroxyphenylacetate 3-monooxygenase, reductase component: hpaC
1.14.14.9
3,4-dihydroxyphenylacetate
homoprotocatechuate 2,3-dioxygenase: hpaD
1.13.11.15
2-hydroxy-5-carboxymethylmuconate semialdehyde
2-hydroxy-5-carboxymethylmuconate semialdehyde dehydrogenase: hpaE
1.2.1.60
5-carboxymethyl-2-hydroxymuconate
5-carboxymethyl-2-hydroxymuconate isomerase: PA14_RS13325
5-carboxymethyl-2-hydroxymuconate isomerase: PA14_RS15870
5-carboxymethyl-2-hydroxymuconate isomerase: PA14_RS04275
5.3.3.10
(3E,5R)-5-carboxy-2-oxohept-3-enedioate
5-carboxymethyl-2-oxo-hex-3-ene-1,7-dioate decarboxylase: PA14_RS13325
4.1.1.68
(4Z)-2-oxohept-4-enedioate
2-oxo-hepta-3-ene-1,7-dioic acid hydratase: hpaH
4.2.1.163
(4S)-4-hydroxy-2-oxoheptanedioate
2,4-dihydroxyhept-2-ene-1,7-dioic acid aldolase: hpaI
4.1.2.52
succinate semialdehyde
NADP+-dependent succinate-semialdehyde dehydrogenase: PA14_RS01425
1.2.1.79
succinate
Branched chain amino acid Biosynthesis
pyruvate
acetolactate synthase small subunit: PA14_RS25400
acetolactate synthase 3 catalytic subunit: ilvB
acetolactate synthase large subunit: PA14_RS03970
2.2.1.6
(S)-2-acetolactate
ketol-acid reductoisomerase: PA14_RS25395
1.1.1.86
(2R)-2,3-dihydroxy-3-methylbutanoate
2,3-dihydroxy-isovalerate dehydratase: PA14_RS01875
4.2.1.9
3-methyl-2-oxobutanoate
2-isopropylmalate synthase: PA14_RS19720
2-isopropylmalate synthase: leuA
2.3.3.13
branched chain amino acid aminotransferase: PA14_RS27050
2.6.1.42
val
(2S)-2-isopropylmalate
3-isopropylmalate dehydratase large subunit: leuC
3-isopropylmalate dehydratase small subunit: leuD
2-isopropylmaleate
3-isopropylmalate dehydratase large subunit: leuC
3-isopropylmalate dehydratase small subunit: leuD
(2R,3S)-3-isopropylmalate
3-isopropylmalate dehydrogenase: leuB
(2S)-2-isopropyl-3-oxosuccinate
4-methyl-2-oxopentanoate
branched chain amino acid aminotransferase: PA14_RS27050
2.6.1.6/2.6.1.42
leu
thr
serine/threonine dehydratase: PA14_RS12000
serine/threonine dehydratase: PA14_RS21685
threonine ammonia-lyase: ilvA
(2Z)-2-aminobut-2-enoate
2-iminobutanoate
3.5.99.-
2-oxobutanoate
acetolactate synthase small subunit: PA14_RS25400
acetolactate synthase 3 catalytic subunit: ilvB
acetolactate synthase large subunit: PA14_RS03970
2.2.1.6
(S)-2-aceto-2-hydroxybutanoate
ketol-acid reductoisomerase PA14_RS25395
1.1.1.86
(R)-2,3-dihydroxy-3-methylpentanoate
2,3-dihydroxy-3-methylvalerate hydro-lyase: PA14_RS01875
4.2.1.9
(S)-3-methyl-2-oxopentanoate
branched chain amino acid aminotransferase: PA14_RS27050
2.6.1.42
ile

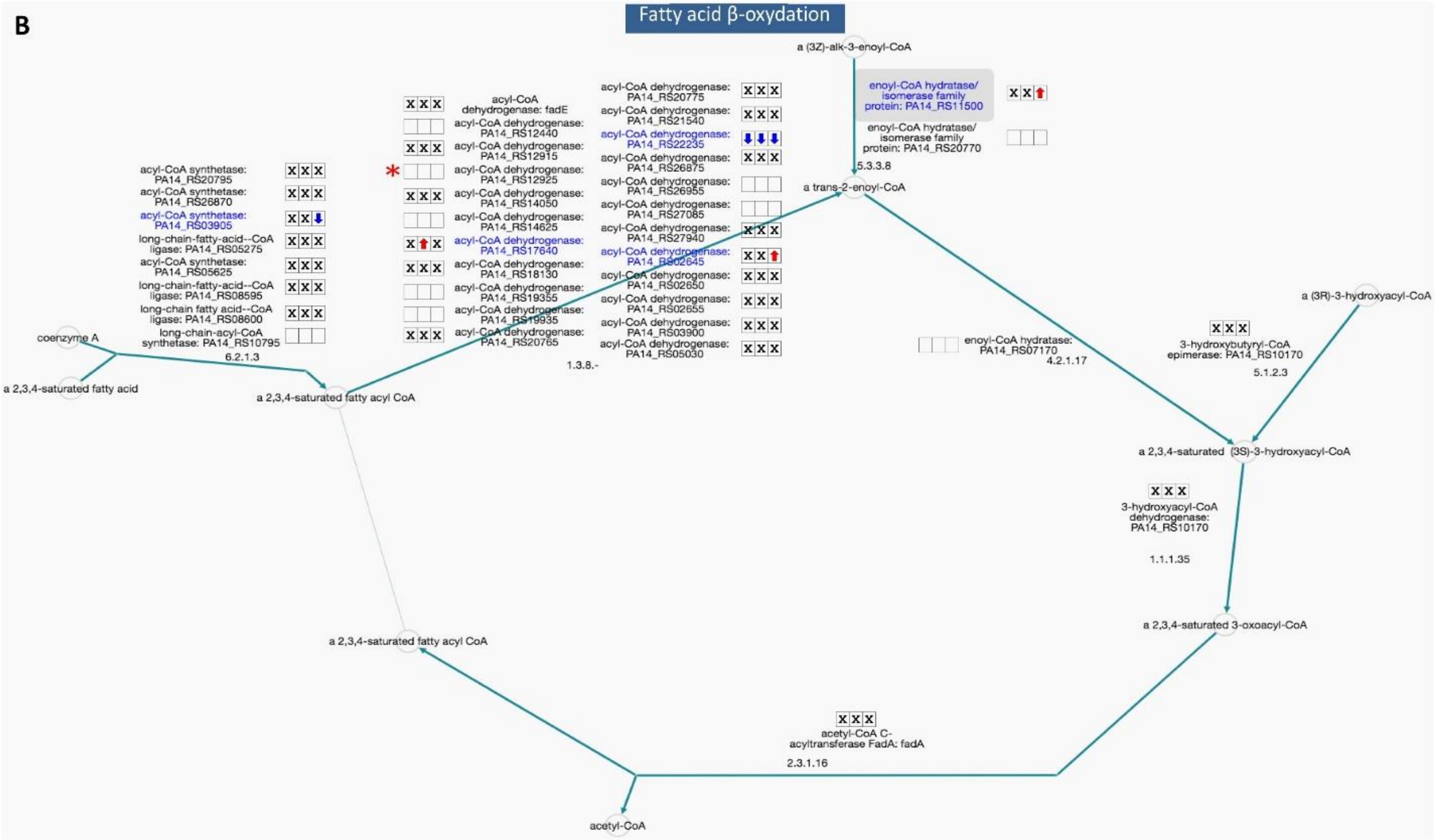
B
Fatty acid β-oxydation
a (3Z)-alk-3-enoyl-CoA
enoyl-CoA hydratase/ isomerase family protein: PA14_RS11500
enoyl-CoA hydratase/ isomerase family protein: PA14_RS20770
5.3.3.8
a trans-2-enoyl-CoA
acyl-CoA dehydrogenase: fadE
acyl-CoA dehydrogenase: PA14_RS12440
acyl-CoA dehydrogenase: PA14_RS12915
acyl-CoA dehydrogenase: PA14_RS12925
acyl-CoA dehydrogenase: PA14_RS14050
acyl-CoA dehydrogenase: PA14_RS14625
acyl-CoA dehydrogenase: PA14_RS17640
acyl-CoA dehydrogenase: PA14_RS18130
acyl-CoA dehydrogenase: PA14_RS19355
acyl-CoA dehydrogenase: PA14_RS19935
acyl-CoA dehydrogenase: PA14_RS20765
acyl-CoA dehydrogenase: PA14_RS20775
acyl-CoA dehydrogenase: PA14_RS21540
acyl-CoA dehydrogenase: PA14_RS22235
acyl-CoA dehydrogenase: PA14_RS26875
acyl-CoA dehydrogenase: PA14_RS26955
acyl-CoA dehydrogenase: PA14_RS27085
acyl-CoA dehydrogenase: PA14_RS27940
acyl-CoA dehydrogenase: PA14_RS02645
acyl-CoA dehydrogenase: PA14_RS02650
acyl-CoA dehydrogenase: PA14_RS02655
acyl-CoA dehydrogenase: PA14_RS03900
acyl-CoA dehydrogenase: PA14_RS05030
1.3.8.-
acyl-CoA synthetase: PA14_RS20795
acyl-CoA synthetase: PA14_RS26870
acyl-CoA synthetase: PA14_RS03905
long-chain-fatty-acid--CoA ligase: PA14_RS05275
acyl-CoA synthetase: PA14_RS05625
long-chain-fatty-acid--CoA ligase: PA14_RS08595
long-chain fatty acid--CoA ligase: PA14_RS08600
long-chain-acyl-CoA synthetase: PA14_RS10795
6.2.1.3
coenzyme A
a 2,3,4-saturated fatty acid
a 2,3,4-saturated fatty acyl CoA
enoyl-CoA hydratase: PA14_RS07170
4.2.1.17
a (3R)-3-hydroxyacyl-CoA
3-hydroxybutyryl-CoA epimerase: PA14_RS10170
5.1.2.3
a 2,3,4-saturated (3S)-3-hydroxyacyl-CoA
3-hydroxyacyl-CoA dehydrogenase: PA14_RS10170
1.1.1.35
a 2,3,4-saturated 3-oxoacyl-CoA
a 2,3,4-saturated fatty acyl CoA
acetyl-CoA C-acyltransferase FadA: fadA
2.3.1.16
acetyl-CoA

Suppl. Figure 4. (A, B). Pathways with core metabolism genes with implications in virulence (red stars) and at least one gene (named in blue) differentially expressed in a consistent manner in comparisons of at least one highly virulent strain to all low virulent strains. All genes are labelled according to their differential expression patterns with boxed symbols: empty – no differential expression; red arrow – Upregulated; blue arrow – downregulated; X – conflicting differential expression. The 3 boxes correspond (left to right) to strains B136-33, MTB-1 and PA14 respectively. The L-leucine biosynthesis pathway is depicted as part of the superpathway of branched chain amino acid biosynthesis.
